# Identification of SMG6 cleavage sites and a preferred RNA cleavage motif by global analysis of endogenous NMD targets in human cells

**DOI:** 10.1093/nar/gku1258

**Published:** 2014-11-27

**Authors:** Skye A. Schmidt, Patricia L. Foley, Dong-Hoon Jeong, Linda A. Rymarquis, Francis Doyle, Scott A. Tenenbaum, Joel G. Belasco, Pamela J. Green

**Affiliations:** 1 Delaware Biotechnology Institute and Department of Plant and Soil Sciences, University of Delaware, Newark, DE 19711, USA; 2 Kimmel Center for Biology and Medicine at the Skirball Institute and Department of Microbiology, New York University School of Medicine, New York, NY 10016, USA; 3 Department of Life Science, Hallym University, Chuncheon, Gangwon, Republic of Korea; 4 College of Nanoscale Science and Engineering, SUNY-Polytechnic Institute, Albany, NY 12203, USA

## Abstract

In metazoans, cleavage by the endoribonuclease SMG6 is often the first degradative event in non-sense-mediated mRNA decay (NMD). However, the exact sites of SMG6 cleavage have yet to be determined for any endogenous targets, and most evidence as to the identity of SMG6 substrates is indirect. Here, we use Parallel Analysis of RNA Ends to specifically identify the 5′ termini of decay intermediates whose production is dependent on SMG6 and the universal NMD factor UPF1. In this manner, the SMG6 cleavage sites in hundreds of endogenous NMD targets in human cells have been mapped at high resolution. In addition, a preferred sequence motif spanning most SMG6 cleavage sites has been discovered and validated by mutational analysis. For many SMG6 substrates, depletion of SMG6 resulted in the accumulation of decapped transcripts, an effect indicative of competition between SMG6-dependent and SMG6-independent NMD pathways. These findings provide key insights into the mechanisms by which mRNAs targeted by NMD are degraded.

## INTRODUCTION

Nonsense-mediated mRNA decay (NMD) is a post-transcriptional surveillance process that recognizes and degrades mRNAs containing premature termination codons (PTCs) in eukaryotes. In addition to aberrant transcripts that have a PTC due to a DNA mutation, an error in transcription, or unproductive splicing, NMD is thought to target 3–10% of normal transcripts in humans (1–4), thereby serving as a widespread gene regulatory mechanism. The biomedical significance of this process is evident from the many inherited and acquired diseases caused by PTCs and the potential for therapies based on PTC readthrough or NMD inhibition (5–9).

The protein machinery of NMD in human cells consists of the factors UPF1, UPF2, UPF3, SMG1, SMG8 and SMG9, which are involved in PTC recognition, as well as SMG5, SMG6, SMG7 and PNRC2, which participate in target degradation (for reviews, see (10–13)). Importantly, with the exception of UPF1, not all of these factors are required for the degradation of every NMD target (14–17) because NMD employs multiple mechanisms for both substrate recognition and degradation.­­

NMD begins with the recognition of PTCs, which are deemed premature because they are flanked downstream by what the cell perceives to be an abnormal 3′ untranslated region (UTR). Although the defining characteristics of such a 3′ UTR have not been fully delineated, the presence of an exon junction more than 50 nucleotides downstream of the termination codon (TC) (18,19) and the distance between the TC and the poly(A) tail (20,21) have both been implicated. When translation termination is judged to have occurred at a PTC, the essential NMD factor UPF1 assembles with UPF2 and UPF3 near the terminating ribosome (22,23). These interactions promote the phosphorylation of UPF1 (24), which in turn recruits the NMD effector protein(s) SMG6, SMG5/SMG7 or SMG5/PNRC2 (25–28). These NMD effectors mediate degradation of the phosphoUPF1-associated transcript by either endonucleolytic cleavage by SMG6 (29,30) or enhanced deadenylation and/or decapping by SMG5/SMG7 or SMG5/PNRC2 (26,31,32). What determines which of these degradation mechanisms predominates for a given transcript remains to be elucidated (16).

Multiple interactions aid recruitment of the endonuclease SMG6 to PTC-containing transcripts. The most important of these involves binding of the 14–3–3 domain of SMG6 to phosphoUPF1 (27,33). SMG6 recruitment is enhanced by additional contacts between two SMG6 sequence motifs and components of the exon junction complex, which help to explain how an exon junction downstream of a PTC can facilitate NMD (24). Once recruited, SMG6 utilizes its PIN domain to cleave NMD-sensitive transcripts endonucleolytically at various locations in the vicinity of the PTC (29,30,34). The resulting 5′- and 3′-terminal RNA fragments are then rapidly degraded exonucleolytically by the exosome and XRN1, respectively. The exact sites of SMG6 cleavage have been mapped for a few reporter transcripts (29,35–38) but have not been determined for any endogenous targets. Moreover, because no global analysis of sites of SMG6 cleavage has been reported, it is not known whether these sites share common sequence characteristics.

Endogenous targets of NMD in humans have been identified by microarray or RNA-Seq analysis of transcripts from cells in which a key NMD factor, such as UPF1, was depleted (1–3). As expected, most of the transcripts found to be affected in these studies were up-regulated when NMD was attenuated. However, a major limitation of this approach is that it does not distinguish between direct and indirect effects. This concern is validated by the observation that many other transcripts examined in these studies were down-regulated upon depletion of NMD factors, even when message half-lives were measured directly and not merely inferred from changes in mRNA concentration (4). The fact that the depletion of such factors could destabilize a large number of mRNAs raises questions as to whether those that are stabilized can all reliably be considered targets of NMD.

A less ambiguous way to identify transcripts targeted for degradation by a particular pathway is to use Parallel Analysis of RNA Ends (PARE) to examine mRNA decay intermediates whose production depends on a protein critical for that pathway. PARE employs high-throughput sequencing to detect and characterize 3′-terminal degradation intermediates bearing a 5′ monophosphate. This and similar RNA degradome approaches were first used to identify miRNA targets in plants, where miRNA-mediated endonucleolytic cleavage is common (39–41), and subsequently in mammalian cells, where such cleavage is far less common (42–44). PARE/degradome analysis has also been used to identify targets of Drosha (42), an endonuclease that preferentially cleaves double-stranded RNAs, or for diverse broader analyses (e.g. (45–51)). However, these technologies have not been employed to identify the biological substrates of endoribonucleases that cleave mRNA within single-stranded regions, often at sites that are difficult to predict.

In this report, we describe the use of PARE to identify hundreds of endogenous SMG6 targets in human cells from the sequences of their cleavage products. Alignment of the cleavage sites revealed a pentameric motif favored by SMG6. Additional observations indicate that cleavage by SMG6 contributes to autoregulation of NMD and that depletion of SMG6 leads to decapping of many mRNAs ordinarily targeted by this endonuclease. By providing insights into the role of SMG6 in NMD, these findings may help to elucidate the down-regulation of some of the numerous PTC-containing alleles that cause disease.

## MATERIALS AND METHODS

Please see Supplementary Methods for additional experimental details.

### Cell culture and RNA interference

For RNA samples used to prepare the PARE, C-PARE and RNA-Seq libraries, HeLa cell lines that stably express the TCRβ68 reporter transcript (29,52) were treated with siXRN1 and either siSMG6, siUPF1 or siGL2 (see Supplementary Methods). For RNA samples used to prepare SPARE libraries, HeLa cells were transiently transfected with one of eight pTCRβ68U variants that expressed TCRβ68 mRNA in addition to the siXRN1 and siGL2.

### Construction of PARE, C-PARE, SPARE and RNA-Seq libraries

PARE libraries were constructed as previously described (39,53) modified for the Illumina HiSeq platform (54). C-PARE libraries were similarly constructed except poly(A)^+^ mRNA was dephosphorylated with calf intestinal alkaline phosphatase (Invitrogen) then decapped using tobacco acid pyrophosphatase (Epicentre) prior to library construction. SPARE libraries were constructed as previously described (55) with modifications described in Supplementary Methods. RNA-Seq libraries were constructed from poly(A)^+^ RNA using the TruSeq RNA sample prep kit (Illumina).

### Bioinformatic analysis

Trimmed PARE and C-PARE sequences were matched to the human genome hg19 (http://hgdownload.cse.ucsc.edu/downloads.html) and Gencode V11 protein-coding transcripts (http://www.gencodegenes.org/releases/11.html) with 1 nt reiterative mismatching, and perfectly matched to TCRβ68 using Bowtie 0.12.7 (56). Abundance from each library was normalized to total genome-matched reads. SPARE libraries were exactly matched to their TCRβ68U sequence and normalized internally. RNA-Seq libraries were matched to a custom hg19+TCRβ68 index using TopHat v2.0.4 (57) with Bowtie1, allowing for 1 mismatch and no novel junctions. SMG6 targets were identified using custom Perl scripts.

### Identification of a SMG6 cleavage site motif

Sequence logos were created from the 10 nt surrounding the PARE MaxSeq using Weblogo 3 (58) with equiprobable nucleotide frequencies for the endogenous SMG6 targets and pipeline controls.

## RESULTS

### Validation of the efficacy of PARE for identifying NMD intermediates

**Figure 1.**
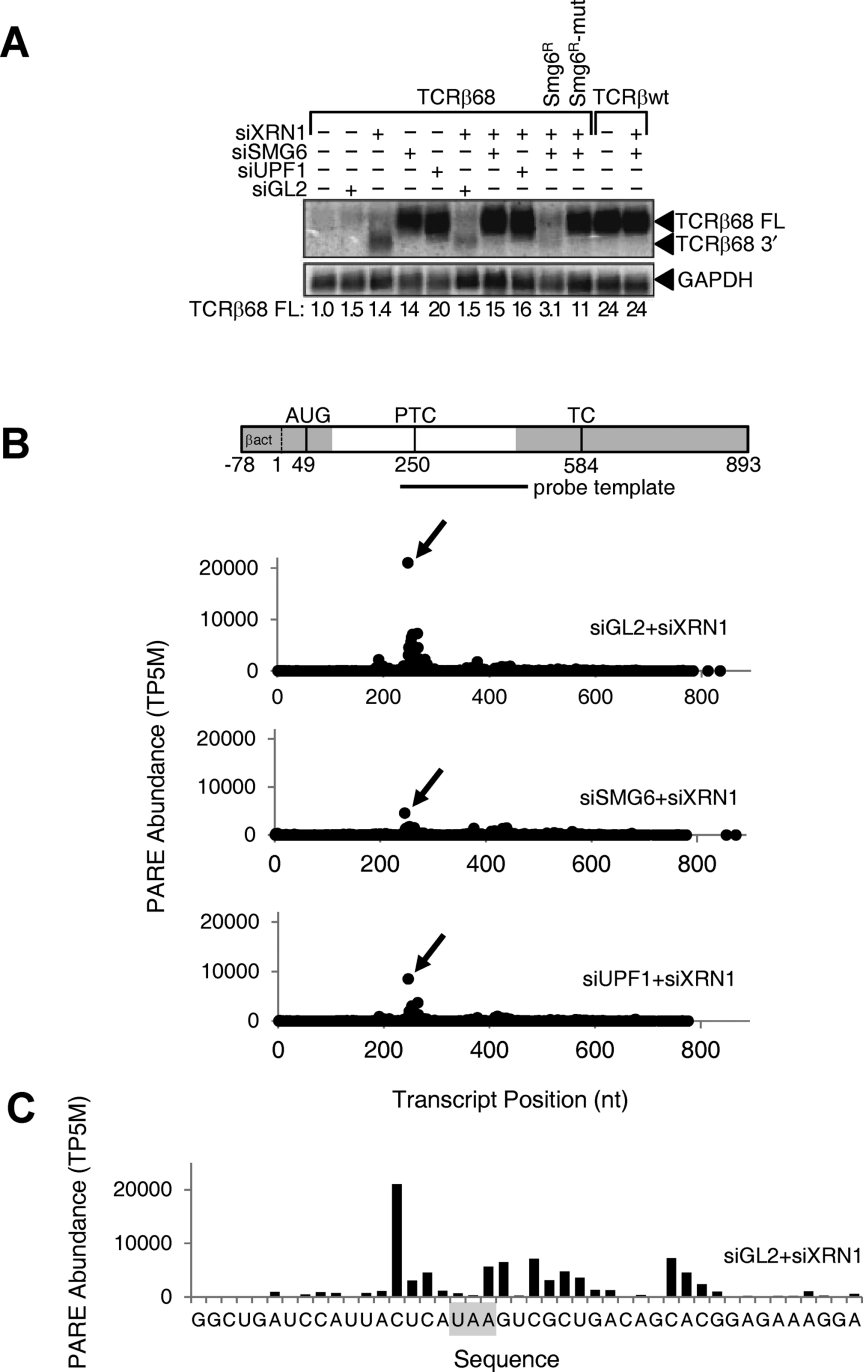
Effect of SMG6 or UPF1 depletion on the concentration of the full-length TCRβ68 transcript and its 3′-terminal SMG6 cleavage product. HeLa cells that expressed a TCRβ68 transcript harboring a PTC at codon 68 (TCRβ68) or a wild-type TCRβ transcript lacking a PTC (TCRβwt) were transfected with siRNAs directed against XRN1, SMG6 or UPF1, or with siGL2 (negative control). Total RNA extracted from these cells was used to prepare PARE libraries. (A) Detection of both a full-length transcript (TCRβ68 FL) and a 3′-terminal decay intermediate (TCRβ68 3′) by northern blot analysis. As a control, some cells were transfected with an siRNA-resistant SMG6 gene (SMG6^R^) or a catalytically inactive variant thereof (SMG6^R^-mut). In each case, the concentration of the full-length transcript relative to that in mock-transfected cells was calculated after normalization to GAPDH mRNA (internal standard). (B) D-plots for the TCRβ68 reporter identifying monophosphorylated 5′ ends detected by PARE in cells transfected with siXRN1 and either siGL2, siSMG6 or siUPF1. A map of the TCRβ68 transcript is shown above the D-plots. Alternating gray and white zones indicate exons. AUG, translation initiation codon; PTC, premature termination codon; TC, natural termination codon. Because the 5′-terminal portion of the reporter was derived from the human β-actin gene, TCRβ68-derived PARE sequences there cannot be distinguished from those originating from the endogenous β-actin transcript. Transcript positions represent the distance from the first nucleotide unique to the TCRβ68 reporter. (C) High-resolution D-plot identifying 5′ ends detected by PARE in RNA from cells transfected with siGL2 and siXRN1. Gray rectangle, PTC.

To identify endogenous targets of SMG6 cleavage by PARE, it was first necessary to define conditions for inhibiting such cleavage by SMG6 depletion and for stabilizing SMG6 cleavage products by XRN1 depletion. For this purpose, we chose a system in which a previously characterized endonucleolytic intermediate of non-sense-mediated degradation accumulates in a SMG6- and UPF1-dependent manner (29). The foundation of this system is a HeLa cell line stably transfected with TCRβ68, a reporter that contains a PTC at codon 68 of the TCRβ transcript (52). Because the PTC in TCRβ68 triggers NMD, the cellular concentration of full-length TCRβ68 mRNA is ordinarily very low and remains low following transfection with a non-specific siRNA (siGL2) (Figure 1A). Depletion of the cytoplasmic 5′ exonuclease XRN1 by transfection with an XRN1-specific siRNA (siXRN1) allows a 3′-terminal fragment of TCRβ68 mRNA to accumulate; otherwise, this decay intermediate is not detectable by northern blotting due to its rapid degradation by XRN1 (29). As expected, when NMD is inhibited by siRNAs that deplete either SMG6 (siSMG6) or UPF1 (siUPF1), full-length TCRβ68 mRNA accumulates to higher levels, and the 3′ cleavage product is no longer visible even in the presence of siXRN1. That this 3′ fragment results from SMG6 cleavage of TCRβ68 mRNA was corroborated by complementation experiments in which cleavage was restored when cells treated with both siXRN1 and siSMG6 were transfected with an siSMG6-resistant gene encoding catalytically active SMG6 (*SMG6^R^*) but not a mutant (*SMG6^R^*-mut) that lacks endonuclease activity (29). Taken together, these data confirm the utility of this HeLa cell system for detecting the downstream product of TCRβ68 mRNA cleavage by SMG6 and, by inference, the corresponding fragment of endogenous SMG6 targets. In particular, 3′-terminal decay intermediates resulting from SMG6 cleavage of endogenous targets should accumulate to higher levels when XRN1 is depleted (transfection with siGL2+siXRN1) but not when SMG6 or UPF1 is also depleted (transfection with siSMG6+siXRN1 or siUPF1+siXRN1).

**Figure 2.**
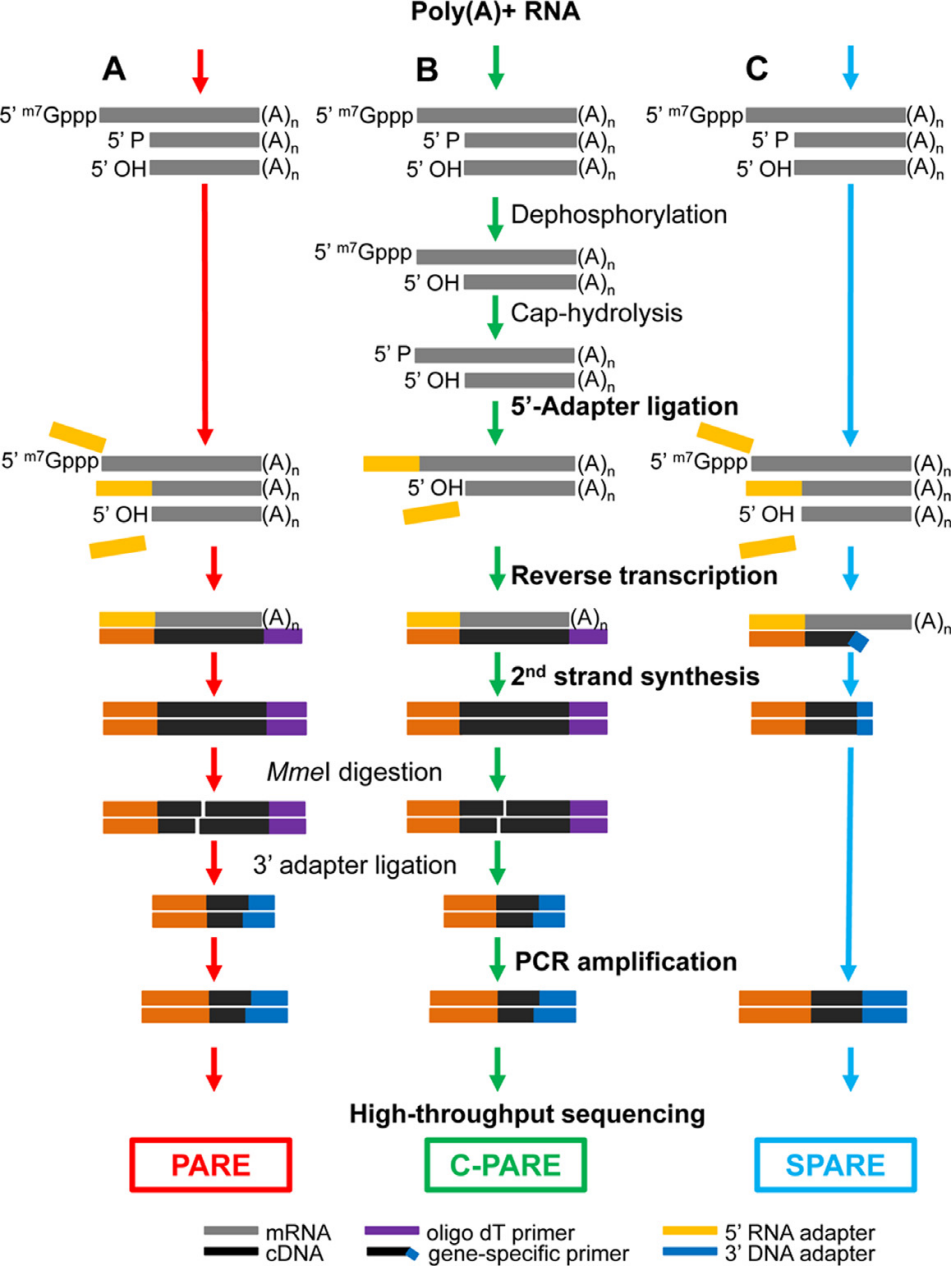
Schematic diagrams for PARE, C-PARE and SPARE library construction. (A) PARE, (B) C-PARE and (C) SPARE libraries were constructed from poly(A)^+^ RNA in multiple steps (red, green or blue arrows). The procedures used to construct all of the libraries are shown in bold lettering, while those used to construct specific libraries are shown in regular lettering. 5′ ^m7^Gppp, capped 5′ end; 5′ P monophosphorylated 5′ end; 5′ OH, hydroxylated 5′ end; (A)_n_, polyadenylated 3′ end.

The 3′-terminal mRNA fragments generated by SMG6 cleavage have a monophosphate at the 5′ end. To characterize this population of RNA decay intermediates, PARE libraries were constructed as shown in Figure 2A, using HeLa RNA from the double knockdown experiments illustrated in Figure 1A. Specifically, PARE libraries capturing monophosphorylated poly(A)^+^ RNAs from HeLa-TCRβ68 cells transfected with siGL2+siXRN1, siSMG6+siXRN1 or siUPF1+siXRN1 were deeply sequenced. A total of 197 230 305 genome-matched PARE sequences from the 5′ ends of these RNAs were obtained from the six PARE libraries, corresponding to 91 943 mRNAs from 20 456 human genes in Gencode V11 (Supplementary Table S1). Averaging almost 9 million distinct genome-matched sequences per library, these replicates provided a rich source of data to identify sites of SMG6 cleavage.

To optimize the design of a bioinformatics pipeline for identifying sites of SMG6 cleavage in endogenous transcripts, we first used PARE to examine the effect of siSMG6 and siUPF1 on the cleavage of the TCRβ68 reporter transcript. Decay plots (D-plots) were constructed by plotting the normalized abundance of the PARE sequences that matched this transcript versus their location (Figure 1B). The 5′ end of the most abundant PARE sequence (MaxSeq) in TCRβ68 was located 4 nt upstream of the PTC (Figure 1C). Compared to the control (siGL2+siXRN1), the abundance of this sequence in both biological replicates decreased by a factor of 4 when SMG6 was depleted and by a factor of 2 when UPF1 was depleted (Figure 1B), suggesting that the PARE MaxSeq represents a prominent site of SMG6 cleavage. These reductions are consistent with northern blot evidence for the decreased accumulation of the TCRβ68 3′ cleavage product in the siSMG6+siXRN1 and siUPF1+siXRN1 samples. In addition to the MaxSeq, other less abundant SMG6- and UPF1-dependent PARE sequences in close proximity to the MaxSeq were also identified in the D-plots (Figure 1B and C). These likely represent additional sites of SMG6 cleavage.

To confirm that PARE accurately identifies the 5′ ends of decay intermediates, we sought to map those ends by other means. 5′ RLM-RACE identified a set of TCRβ68 decay intermediates similar to those observed by PARE (Supplementary Figure S1). To verify the sites of TCRβ68 cleavage by a distinct method that did not involve oligoribonucleotide ligation and reverse transcription-polymerase chain reaction, their location was also mapped by northern blot analysis of mRNA fragments generated by deoxyribozyme (DNAzyme) cleavage. 10–23 DNAzymes are catalytic DNA molecules that efficiently cleave RNA substrates in a site-specific manner, as determined by sequence complementarity to the DNAzyme. RNA from HeLa-TCRβ68 cells transfected with siXRN1 was incubated with a DNAzyme (DZ384) designed to cleave TCRβ68 RNA 138 nt downstream of the putative SMG6 cleavage site represented by the PARE MaxSeq. In addition, to generate a cognate set of size markers, RNA from cells treated with siSMG6 was incubated with both DZ384 and any of several other DNAzymes designed to cleave TCRβ68 mRNA in the vicinity of the 5′ end of the PARE MaxSeq. The electrophoretic mobility of the RNA fragments generated by DNAzyme cleavage of the TCRβ68 decay intermediates, delimited by SMG6 cleavage at their 5′ ends and DZ384 cleavage at their 3′ ends, were examined by northern blot analysis alongside TCRβ68 fragments generated by dual DNAzyme cleavage of full-length TCRβ68 mRNA (Supplementary Figure S1D). Three SMG6 cleavage products were apparent when XRN1 was depleted, each corresponding to cleavage at a site identified both by PARE and by 5′ RLM-RACE. One of these sites coincided with the 5′ end of the PARE MaxSeq, while the other two bands corresponded to other prominent PARE sequences. These results validate the reliability of PARE for accurately identifying sites of SMG6 cleavage in cellular transcripts and corroborate the PARE evidence for SMG6 cleavage at multiple sites within a localized region near the PTC of TCRβ68 mRNA.

### RNA-Seq identification of transcripts negatively regulated by SMG6

The increased abundance of the full-length TCRβ68 transcript upon SMG6 or UPF1 depletion (Figure 1) suggested that at least some endogenous SMG6 targets should behave similarly, particularly those for which SMG6 has a major impact on the rate of decay. To identify those transcripts, RNA-Seq libraries were constructed from the same RNA samples used to make the PARE libraries (Supplementary Table S1). A total of 143 487 780 genome- or transcriptome-matched reads from six libraries were obtained. To limit our analyses to transcript isoforms that are expressed at measurable levels, only the 34 086 endogenous mRNAs that had an average abundance >1.00 FPKM (Fragments Per Kilobase of transcript per Million reads) in the siSMG6+siXRN1 libraries were examined. While the majority of endogenous transcripts were unaffected by NMD factor depletion (Supplementary Figure S2A and B), 15% (5273 transcripts) were significantly elevated when either SMG6 or UPF1 was depleted (*P* < 0.05, Supplementary Figure S2C). A majority of the transcripts elevated in the siSMG6+siXRN1 library were also elevated in the siUPF1+siXRN1 library and vice versa, with a high correlation between the fold changes in each (Supplementary Figure S2D). This analysis identified 2090 mRNAs that were significantly more abundant in both siSMG6+siXRN1 and siUPF1+siXRN1 knockdown libraries than in siGL2+siXRN1 knockdown libraries as potentially the transcripts most impacted by NMD. An additional 6% (2061) of the transcripts were significantly diminished in the siSMG6+siXRN1 library, the siUPF1+siXRN1 library or both; these may be mRNAs whose concentration is subject to secondary effects of NMD (Supplementary Figure S2C). The RNA-Seq reads that matched to the TCRβ68 reporter were also analyzed. Consistent with the northern blot data, the abundance of the TCRβ68 transcript was markedly higher in both the siSMG6+siXRN1 and siUPF1+siXRN1 RNA-Seq libraries as compared to the control siGL2+siXRN1 library (Supplementary Figure S2E).

### Transcriptome-wide identification of SMG6 cleavage sites by PARE and RNA-Seq

**Figure 3.**
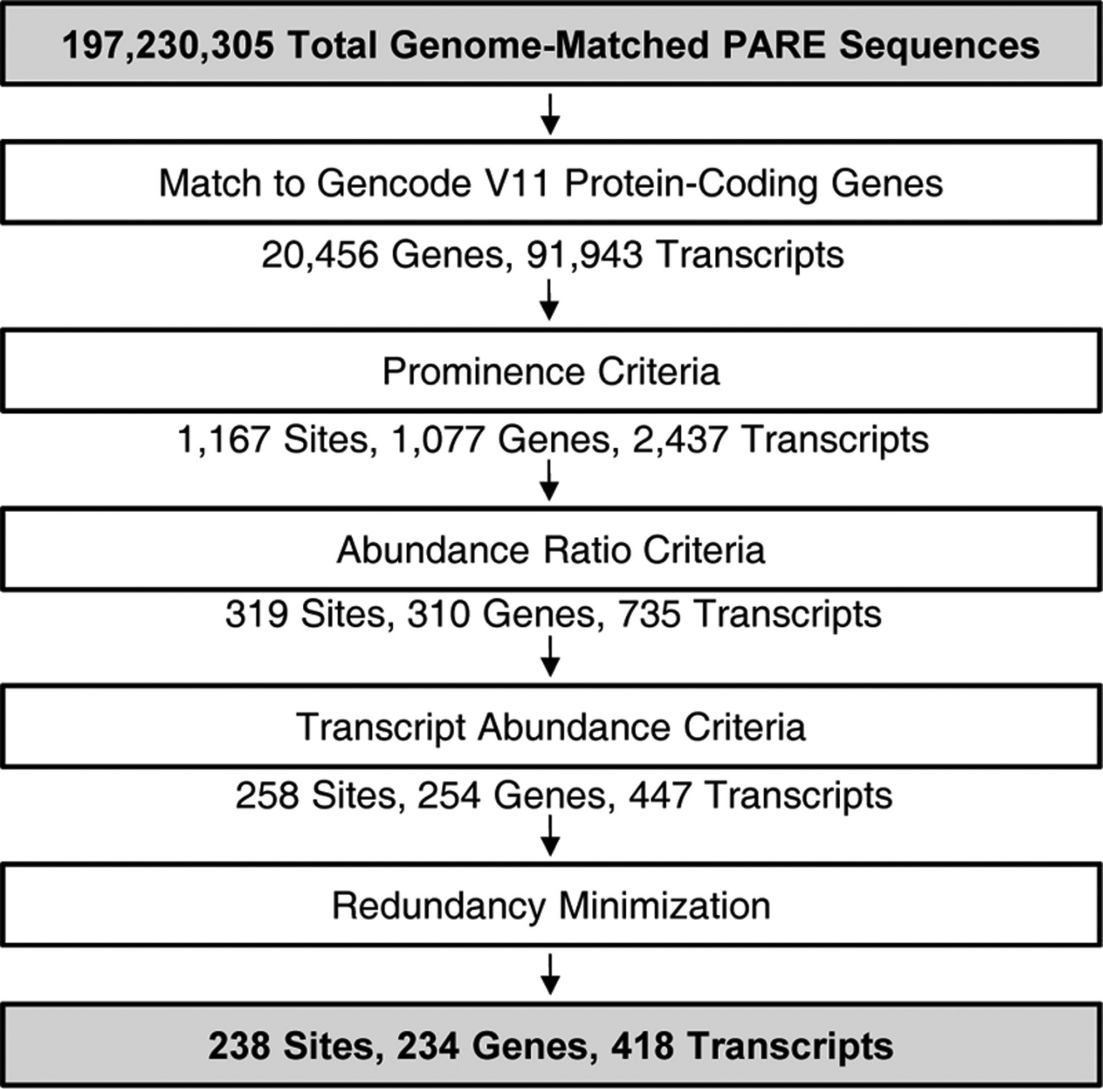
Computational pipeline for identifying SMG6 targets. Input and final output are highlighted in gray. The data were filtered in five steps described in detail in Supplementary Methods. Site refers to a unique PARE MaxSeq in a gene. The pipeline did not include any step intended to favor the presence of a TC near the PARE MaxSeq.

Using the PARE and RNA-Seq libraries, a pipeline comprising five filtering steps was designed to identify endogenous SMG6 targets (Figure 3, described in detail in the Supplementary Methods). The first step simply filtered the genome-matched PARE sequences for those that matched mRNAs. In the second filter step, a set of stringent criteria that selected for prominent and reproducible PARE sequences was designed based on the features of TCRβ68 cleavage by SMG6. Because a single gene can encode multiple transcripts due to alternative processing, different transcript variants with the same PARE MaxSeq were represented in the pipeline. Also, the MaxSeq of a single gene sometimes differs between two transcript variants. To avoid redundancy in the first case and include different variants in the second case, a PARE ‘site’ was defined as the 5′ end of a distinct PARE sequence within a gene (not within a transcript). A third filter selecting for abundance ratio criteria identified PARE sites whose MaxSeq decreased by at least 50% both when SMG6 was depleted and when UPF1 was depleted.

**Figure 4.**
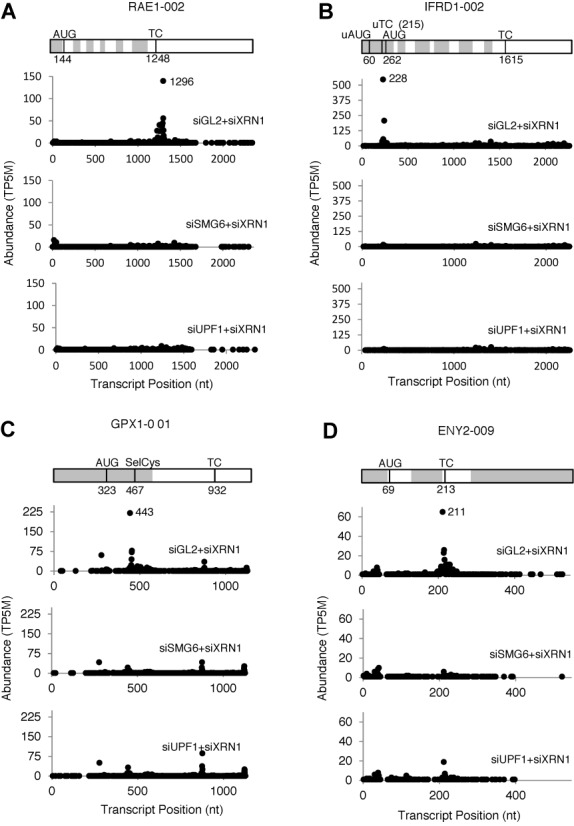
Representative D-plots of endogenous SMG6 targets identified by PARE. A transcript map is shown above each set of D-plots. Alternating gray and white zones represent exons. AUG, translation initiation codon; TC, natural termination codon; uAUG, predicted translation initiation codon of an upstream ORF; uTC, predicted termination codon of an upstream ORF; SelCys, selenocysteine codon. (A) RAE1–002, a transcript with a long 3′ UTR. (B) IFRD1–001, a transcript with a predicted uORF. (C) GPX1–001, a transcript with a selenocysteine codon. (D) ENY2–009, a transcript with an exon junction downstream of the TC. All of the D-plots were drawn with data from Biorep 1; similar effects were observed for Biorep 2.

**Figure 5.**
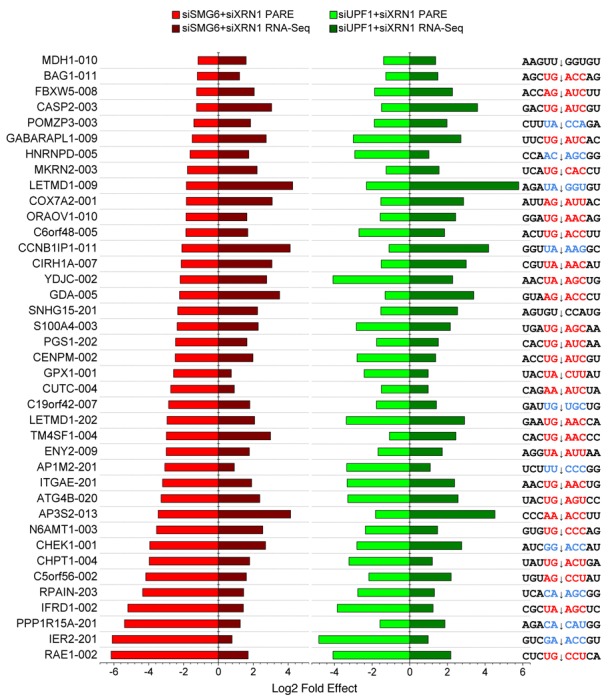
Selected human transcripts targeted by SMG6. Transcripts whose PARE MaxSeq decreased by at least 50% and whose mRNA abundance increased significantly (Cuffdiff *q* < 0.05) in both the siSMG6+siXRN1 and siUPF1+siXRN1 libraries as compared to the siGL2+siXRN1 control are shown. In each case, the magnitude of the effect is represented by a horizontal bar, and the sequence surrounding the principal site of SMG6 cleavage (arrow) is shown. Sequences conforming to the (U/A)-(G/A)-(A/C)-N-(C/U) motif are indicated in red, while sequences with one mismatch are indicated in blue.

The fourth filter step, removing transcripts whose cellular concentration was significantly higher in the siGL2+siXRN1 RNA-Seq libraries, was designed to minimize the influence of RNA accumulation due to secondary effects of NMD disruption. After a final step, in which the PARE data were filtered to minimize ambiguity due to multiple genome hits, a total of 238 PARE sites in 418 transcripts from 234 genes fulfilled all criteria (Supplementary Dataset S1) and were classified as SMG6 cleavage sites. Representative D-plots for several of these illustrate the reproducibility of SMG6-dependent cleavage patterns in biological replicates (Supplementary Figure S3) and provide clear evidence for SMG6 cleavage of transcripts bearing various potential NMD triggers (Figure 4). Of the 238 PARE sites identified in this study, 16% (39 sites) exhibited both a decrease in the concentration of the 3′ cleavage product and a significant increase in overall transcript abundance upon SMG6 depletion and upon UPF1 depletion (Figure 5). For these transcripts, cleavage by SMG6 is likely to be an important mechanism by which their cellular concentration is regulated.

To investigate whether SMG6 targets are functionally related, we used the online DAVID bioinformatics analysis platform (59) to compare the 234 genes identified as SMG6 targets to all expressed genes. This analysis revealed that several functional categories related to oxidative stress and cell death were enriched in our target set (Supplementary Table S2, *P*-value < 0.01). These results support emerging evidence that NMD may play a role in regulating mRNA levels in response to stress (60–62).

Several NMD factors are thought to be subject to feedback regulation by NMD (3,63). However, due to the potential for secondary effects of NMD and the multiple pathways by which NMD can occur, it is not yet known whether SMG6 cleaves any mRNAs that encode NMD factors. To address this question, we analyzed the D-plots of ten SMG- and UPF-family genes involved in NMD (Supplementary Figure S4). The pipeline unambiguously identified UPF3B as a target of SMG6 cleavage with a MaxSeq at position 1020 nt (Supplementary Figure S4J). In addition, SMG1, SMG9 and UPF2 had PARE MaxSeqs consistent with SMG6 cleavage but did not meet all of the criteria of the pipeline. For the remaining genes (SMG5, SMG6, SMG7, SMG8, UPF1 and UPF3A), no prominent site of SMG6-dependent cleavage was observed in any isoform, despite expression levels greater than the pipeline threshold. Together, these results indicate that NMD is indeed autoregulated by SMG6 cleavage in a transcript-preferential manner.

### Identification of a sequence motif enriched at SMG6 cleavage sites

**Figure 6.**
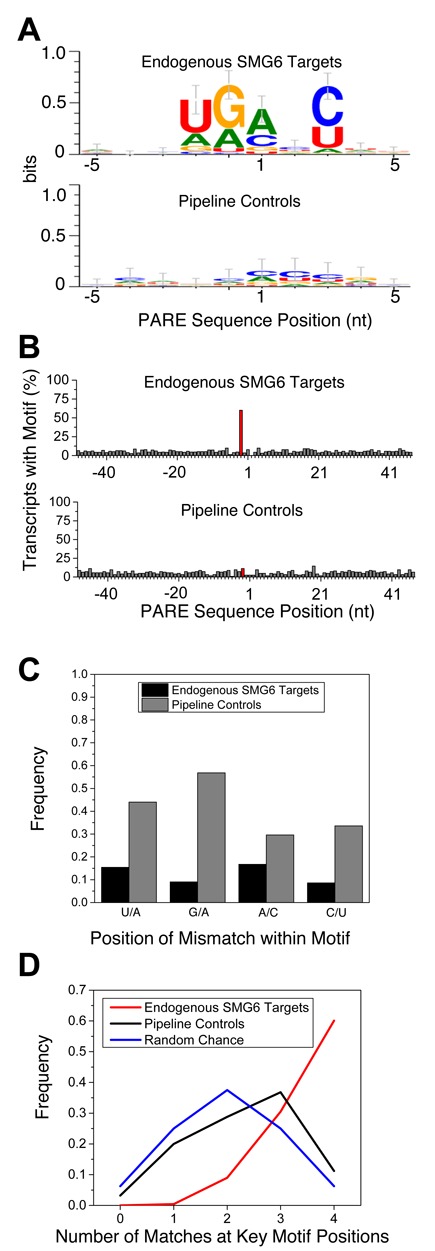
Degenerate pentameric motif common at SMG6 cleavage sites. (A) Sequence logo representation of the 10-nt region surrounding the PARE MaxSeq in endogenous SMG6 targets and siGL2+siXRN1 pipeline controls. (B) Frequency of the 5-nt motif at each position in SMG6 targets and control transcripts. The red bar indicates where the motif would be expected to start in SMG6 targets, 2 nt upstream from the cleavage site. (C) Mismatch frequency at various positions within PARE sites that conform to the SMG6 motif at all but one position. (D) Frequency of SMG6 targets and pipeline controls whose MaxSeq is surrounded by a pentamer that matches the SMG6 motif at 0–4 of the defined positions, versus the frequency that such matches would occur by chance.

In view of the PARE evidence that not all positions near a putative NMD signal are equally susceptible to SMG6 cleavage, we hypothesized that SMG6 may have target site preferences. To determine whether SMG6 cleavage sites share common sequence characteristics, we generated sequence logos (64) of the 10-nt region surrounding the 238 SMG6 cleavage sites. This analysis identified a degenerate pentameric sequence motif, (U/A)-(G/A)↓(A/C)-N-(C/U) straddling the PARE MaxSeq in over 60% of SMG6 targets (Figure 6A and B). An additional 30% of SMG6 targets matched the motif at all but one position (see Supplementary Dataset S1), with no bias observed in the location of the mismatch within the 5-nt motif (Figure 6C). The frequency of the motif in a pipeline control set of SMG6-independent 5′ ends was significantly lower and much closer to that expected by chance (Figure 6A and D). Furthermore, when 100 nt flanking the PARE MaxSeq was analyzed, the frequency of the motif was found to be elevated only at a position starting 2 nt upstream of the PARE MaxSeq (Figure 6B).

To rule out the possibility that the motif might be due to SMG6-independent sequence bias in the siGL2+siXRN1 libraries, we selectively removed or enriched PARE sequences on the basis of fold change in the siSMG6+siXRN1 and siUPF1+siXRN1 libraries. Any resemblance to the motif was lost as PARE sequences with higher fold changes were removed (Supplementary Figure S5A). Conversely, the motif was clearly evident when ratios greater than 2- to 5-fold were enriched (Supplementary Figure S5B). Together with the fact that the first two nucleotides of the conserved motif precede the 5′ end of the decay intermediates detected by PARE, these findings argue against the possibilities that the motif might merely be a signal for XRN1 pausing or an artifact of PARE analysis.

To test the significance of the SMG6 cleavage motif, we generated a set of six TCRβ68 reporters in which the five nucleotides surrounding the MaxSeq of TCRβ68 were varied to create alternative pentamers predicted to be either favorable or unfavorable for SMG6 cleavage. HeLa cells were transiently transfected with these reporter genes, and the 3′-terminal RNA fragments resulting from SMG6 cleavage of each TCRβ68 variant were analyzed by SPARE (55), a transcript-specific adaptation of PARE (Figure 2C).

**Figure 7.**
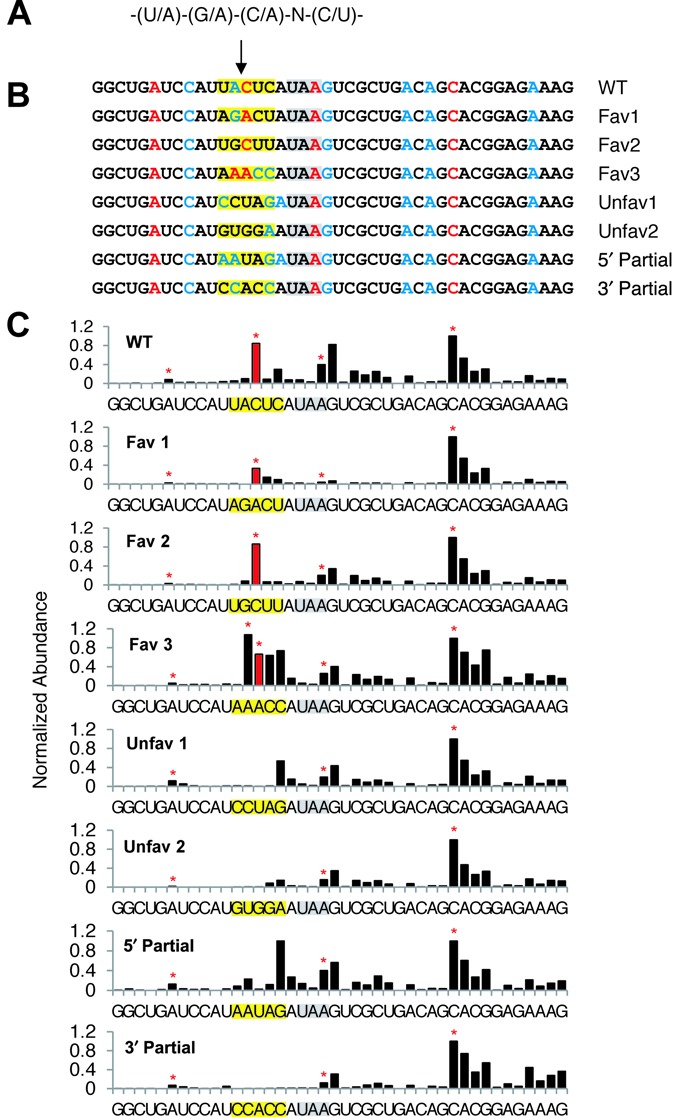
SPARE analysis of the pentameric motif. (A) The degenerate sequence of the motif. (B) PTC-proximal sequences of the SPARE reporters. The SMG6 cleavage site under investigation is marked by an arrow. The PTC is highlighted in gray. The altered motif sequences are highlighted in yellow. Red or blue letters identify the 5′ end of the expected cleavage products for sequence pentamers that matched the motif at every position (at 4 out of 5 positions). (C) SPARE D-plots. The bar heights in each D-plot were normalized to that of the abundant 5′ end 12 nucleotides downstream of the PTC. The red bar identifies the 5′ end resulting from cleavage at the PARE MaxSeq for the original TCRβ68 reporter. Red stars indicate the expected 5′-end generated by cleavage in pentamers that matched the motif at every position.

The SPARE results from two biological replicates (Figure 7 and Supplementary Figure S6) were consistent with each other and with the PARE analysis of TCRβ68 described above (Figure 1). As expected, the TCRβ68 transcripts were all cleaved in close proximity to the PTC, generating a number of closely spaced 5′ ends (Figure 7). The location of the ends observed for the wild-type transcript was the same when examined by either PARE or SPARE, although their relative abundance differed somewhat, most likely due to positional bias arising from the close proximity of the transcript-specific primer used for reverse transcription in the SPARE experiments. Importantly, cleavage was prominent at the same position as the previously identified MaxSeq for each of the four variants that bore a favorable SMG6 motif there (Fav 1–3). Conversely, little or no cleavage was evident at that location for either of the two reporters in which the sequence there was unfavorable for SMG6 (Unfav 1–2). In all six variants, additional SMG6 cleavage sites were present. These generally coincided with nearby sequences that either matched the canonical SMG6 cleavage motif or differed from this motif at only one position. Besides the three canonical motifs that coincided with a prominent site of cleavage, there was a fourth such motif 12 nt upstream of the PTC that did not, suggesting that the context of a SMG6 motif may affect its utilization.

These results suggest that SMG6 cleavage preferentially occurs within a favorable sequence context but can also occur less frequently at other locations, and that the likelihood of cleavage at any particular position depends, in part, on how closely the surrounding pentamer resembles a favorable motif. To evaluate the contribution of the portions of the SMG6 motif that precede or follow the cleavage site, we designed two additional TCRβ68 reporters containing hybrid pentamers that bore favorable nucleotides at positions upstream of the MaxSeq and unfavorable nucleotides downstream of the MaxSeq, or vice versa (5′ Partial, 3′ Partial). SMG6-mediated cleavage at the location of the original MaxSeq was not observed for either of these hybrids by SPARE, indicating that neither the upstream nor downstream portion of the motif is alone sufficient to promote cleavage at that position.

### Accumulation of decapped degradation intermediates upon SMG6 depletion

**Figure 8.**
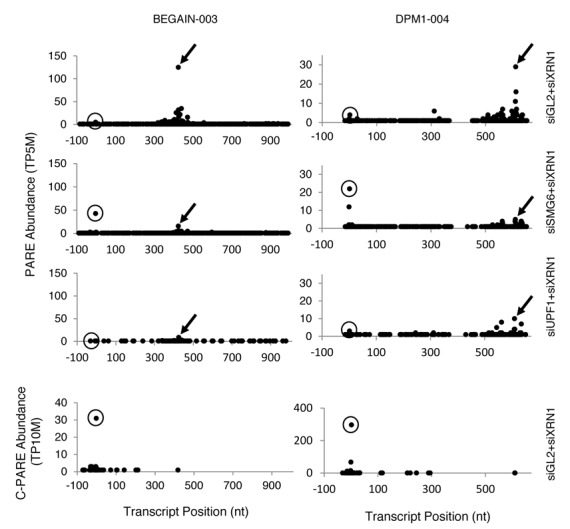
Accumulation of decapped transcripts when SMG6 but not UPF1 is depleted. Arrows in the D-plots of representative transcripts (BEGAIN-003 and DPM1–004) in Biorep 1 identify 3′ cleavage products whose accumulation is SMG6- and UPF1-dependent. Circles indicate the principal location of the 5′ cap, as determined by C-PARE (bottom panel). Similar effects were observed in Biorep 2.

For several endogenous SMG6 targets, the abundance of a PARE sequence near the annotated transcription initiation site (TSS) was observed to increase in siSMG6+siXRN1 cells, but not siUPF1+siXRN1 cells, relative to the siGL2+siXRN1 control (Figure 8). Since NMD is known to proceed by either SMG6-mediated endonucleolytic cleavage or by deadenylation and/or decapping, we hypothesized that these PARE sequences may represent an increased presence of decapped transcripts upon SMG6 depletion. To determine how close these SMG6-dependent PARE sequences in the 5′ region were to the cap, Cap PARE (C-PARE) libraries were generated by treating the same polyadenylated RNA used for PARE first with alkaline phosphatase and then with tobacco acid pyrophosphatase (Figure 2B and Supplementary Table S1). Consequently, C-PARE selectively captures the 5′-terminal sequences of polyadenylated RNAs whose 5′ phosphates are protected by a cap. These C-PARE sequences were compared to the sequences of mRNAs beginning 100 nt upstream of the TSS. The upstream region was included to capture nearby cap sites that were not annotated accurately or are differentially utilized in HeLa cells.

The 5′-terminal sequences of the decay intermediates identified by PARE were also matched to gene sequences beginning 100 nt upstream of the annotated TSS. To find transcripts for which a monophosphorylated 5′ end proximal to the cap was elevated in cells treated with siSMG6+siXRN1, transcripts were selected in which the most abundant PARE sequence within 25 nt of the annotated TSS was in the same position in both biological replicates, was elevated at least 1.5-fold over siGL2+siXRN1, and had a corresponding sequence in the C-PARE libraries whose abundance was at least 5 TP10M. A total of 83 transcripts from 55 genes (∼25% of the SMG6 target genes) were identified in which a decay intermediate accumulated at the cap position upon SMG6 depletion. Depletion of UPF1 did not cause a similar accumulation of this intermediate. Therefore, the increased abundance of decapped transcripts is specific to the depletion of SMG6 rather than the inhibition of NMD.

## DISCUSSION

Endonucleolytic cleavage by SMG6 is an important mechanism for initiating nonsense-mediated degradation of both aberrant and natural transcripts in metazoan cells. In this study, we used PARE to map the sites of SMG6 cleavage in endogenous human mRNAs on a transcriptome-wide scale at single-nucleotide resolution. This strategy made it possible to identify a sequence motif that spans most SMG6 cleavage sites. The importance of the motif was demonstrated by mutational analysis showing that cleavage by SMG6 is markedly enhanced by a favorable sequence context. Interestingly, for many SMG6 targets, decapped decay intermediates were observed to accumulate upon SMG6 depletion, evidence that SMG6 cleavage competes with alternative NMD initiation mechanisms that involve decapping. These advances were made possible by the high sensitivity and global reach of PARE. Beyond these findings, the rich data sets from this report should facilitate future research on SMG6, UPF1 and NMD.

By mapping sites of SMG6 cleavage, we have identified hundreds of endogenous transcripts that are targeted by this endonuclease. This list is not likely to be exhaustive because of the highly stringent criteria that were used to identify SMG6 targets. Prior to this work, none of the cleavage sites within endogenous SMG6 targets had been precisely mapped, and reports documenting the cleavage of native mRNAs by SMG6 were scarce. The ability of PARE to directly capture and sequence decay intermediates is in contrast to previous transcriptome studies that utilized microarray analysis or RNA-Seq to identify mRNAs whose concentration may be influenced by NMD either directly or indirectly. As judged by RNA-Seq, the cellular concentration of only a subset of the SMG6 targets identified by PARE increased when either SMG6 or UPF1 was depleted (Figure 5). The fact that the abundance of the majority of transcripts that we identified as SMG6 targets did not change significantly strongly suggests that many *bona fide* targets would be missed by relying on changes in RNA abundance as the principal criterion. Therefore, an increase in transcript abundance upon SMG6 depletion may not be an accurate predictor of direct susceptibility to NMD. Indeed, of the 234 genes whose transcript(s) we identified as targets of SMG6, only about 10% (23 genes) have previously been classified as NMD targets (1–4). Consistent with our results, a prior study found that only 10% of transcripts whose half-lives were prolonged by UPF1 depletion also increased in cellular concentration (4).

While the abundance of several mRNAs encoding NMD factors changed when SMG6 was depleted, PARE made it possible to delineate which are targets of SMG6 cleavage, a key insight for resolving the complex mechanism of NMD autoregulation. The most prominent such phenotype was observed for UPF3B, which besides being cleaved by SMG6 has also been implicated in the autoregulation of mammalian UPF and SMG transcripts (63). Moreover, loss of function UPF3B mutations in humans not only cause deficiencies in NMD but also lead to a variety of mental disorders that highlight the importance of UPF3B for human health (65–67). The UPF2, SMG1 and SMG9 transcripts were also identified by PARE as potential targets of SMG6 cleavage. By contrast, SMG5 mRNA did not come through our target pipeline, nor could we categorize it as a potential target because the signals were so close to background. On this point it is worth noting that, although the 5′ ends of a number of SMG5 mRNA decay intermediates have recently been mapped by sequencing, the production of those intermediates was not shown to be SMG6-dependent (38); therefore, whether this transcript is a target of SMG6 cleavage remains uncertain. The prominent cleavage by SMG6 of mRNAs encoding a few NMD factors, while the cellular concentration of others not demonstrably cleaved by SMG6 nevertheless increases upon attenuating NMD, raises the possibility that NMD autoregulation could occur in part through downstream effects of the cleavage of a few key UPF/SMG transcripts. Alternatively, mRNAs encoding other NMD factors may be degraded by an NMD mechanism that involves deadenylation and/or decapping.

Examination of the mRNA sequence surrounding the 5′ end of the most abundant decay intermediate observed for each SMG6 target revealed that cleavage usually occurs within the degenerate pentameric motif (U/A)-(G/A)↓(A/C)-N-(C/U). Although this motif would occur at random only 6.25% of the time, more than 60% of the principal SMG6 cleavage sites detected by PARE coincided with such a sequence in its entirety, and 90% of them matched the motif at all or all but one position. The sequence motif, which begins two nucleotides upstream of the SMG6 cleavage site, is not enriched at the 5′ ends of SMG6-independent decay intermediates. Moreover, because most of the SMG6-dependent cleavage sites are not located inside an in-frame TC and only about half contain a UGA or UAA triplet, it is not likely that the first three nucleotides of the motif reflect a preference for SMG6 cleavage within a TC.

To investigate further the importance of the pentameric motif for SMG6 cleavage, we analyzed the cleavage pattern for a number of TCRβ68 transcript variants in which the sequence surrounding the principal SMG6 cleavage site was replaced with pentamers predicted to be favorable or unfavorable for cleavage. We found that cleavage occurred at the predicted site when a favorable motif was substituted at that position, whereas cleavage there was greatly diminished when such a motif was absent. In one of the variants, only the first two nucleotides of a favorable pentamer (AA↓ACC) were changed to an unfavorable sequence (CC↓ACC), yet this substitution virtually abolished cleavage there despite retention of a trimeric sequence often found at the 5′ end of SMG6 cleavage products. This and other evidence for the critical importance of the two nucleotides that are immediately upstream of a potential cleavage site (and therefore absent from the sequenced cleavage product) makes it unlikely that the SMG6-dependent detection of 5′ ends at favorable sites can be attributed to factors such as ligation efficiency, sequencing bias, or XRN1 impediments. Instead, our findings support the conclusion that the pentameric motif in its entirety is characteristic of sites susceptible to SMG6 cleavage.

To gain further insight into the features that cause endogenous transcripts to be targeted by SMG6, we examined the location of the SMG6 cleavage sites relative to TCs that might trigger NMD. For each SMG6 cleavage site, we computationally identified the annotated transcript variant in which a potential NMD-inducing TC was in closest proximity. For about half of the SMG6 cleavage sites, a potential NMD trigger corresponding to the TC of an upstream open reading frame (uORF), a UGA triplet encoding selenocysteine (SelCys), or a natural TC 5′ to a downstream exon junction (dEJ) was present nearby. When a TC with the potential to trigger NMD could be discerned, the principal site of SMG6 cleavage usually coincided with a neighboring (U/A)-(G/A)↓(A/C)-N-(C/U) motif (Supplementary Figure S7). We analyzed the other half of the SMG6 targets to determine whether their cleavage might be triggered by inefficient translation termination due to an unusually long 3′ UTR (3,4,20,68). Although we found that SMG6 targets have longer 3′ UTRs on average than non-targets, the annotated 3′ UTR length was similar for transcripts containing or lacking a predicted NMD trigger (Supplementary Figure S7D). Moreover, a substantial majority of the cleavage sites for which we could not identify a potential trigger coincided with a favorable SMG6 motif well upstream of the TC (Supplementary Figure S7A) and therefore could not be attributed to a long 3′ UTR. These findings suggest that many such SMG6 cleavage products may arise from targeting splice variants or, alternatively, from cleaving transcripts containing cryptic features that trigger NMD by an unconventional mechanism.

In addition to endonucleolytic cleavage by SMG6, NMD can also begin with deadenylation and/or decapping mediated by SMG5/SMG7 or SMG5/PNRC2 (32,69). Our analyses of poly(A)^+^ RNA by PARE do not reveal which of these NMD mechanisms generally predominates. Nevertheless, our discovery that many SMG6 targets accumulate a decapped intermediate when SMG6, but not UPF1, is depleted suggests that transcripts ordinarily cleaved by SMG6 can be degraded by an alternative NMD mechanism when SMG6 is scarce. This evidence for competition among NMD effectors is consistent with recent reports that the effects of SMG6 depletion can be exacerbated by co-depletion of the NMD factor SMG7 (16,28,69).

PARE has proven to be an extraordinarily effective method for mapping sites of SMG6 cleavage and thereby identifying the cellular targets in which they reside. In addition, this strategy has yielded new insights into NMD by identifying a sequence motif characteristic of most SMG6 cleavage sites and by providing evidence for competition among NMD mechanisms. Now that the sites of SMG6 cleavage have been mapped on a global scale, they can be correlated with single nucleotide polymorphisms and other natural mutations, and the consequences of their mutation can be investigated. We expect this overall approach to be equally useful for rapidly characterizing the specificity and mechanistic aspects of other key endonucleases that govern the degradation of transcripts important for human health and disease. Furthermore, the PARE data sets generated in the course of these studies represent a unique and rich resource that can be further mined for additional insights into the action of SMG6 and UPF1 in this and other cellular processes in which they have been implicated (70–72).

## ACCESSION NUMBERS

All RNA-Seq, PARE, SPARE and C-PARE data files will be available for download from NCBI Gene Expression Omnibus (http://www.ncbi.nlm.nih.gov/geo) under accession number GSE61398.

## SUPPLEMENTARY DATA

Supplementary Data are available at NAR Online.

SUPPLEMENTARY DATA
