# Supplementary material for: Identification of SMG6 cleavage sites and a preferred RNA cleavage motif by global analysis of endogenous NMD targets in human cells
Source: Nucleic Acids Res. 2014 Nov 27;43(1):309–23. doi: 10.1093/nar/gku1258 (PMC4288159; doi:10.1093/nar/gku1258)
Supplement: SUPPLEMENTARY DATA [file supp_43_1_309__index.html]

Identification of SMG6 cleavage sites and a preferred RNA cleavage motif by global analysis of endogenous NMD targets in human cells — SUPPLEMENTARY DATA 

# Identification of SMG6 cleavage sites and a preferred RNA cleavage motif by global analysis of endogenous NMD targets in human cells

## SUPPLEMENTARY DATA

**Files in this Data Supplement:**

- SUPPLEMENTARY DATA
- SUPPLEMENTARY DATA
