# Supplementary material for: Identification of SMG6 cleavage sites and a preferred RNA cleavage motif by global analysis of endogenous NMD targets in human cells
Source: Nucleic Acids Res. 2014 Nov 27;43(1):309–23. doi: 10.1093/nar/gku1258 (PMC4288159; doi:10.1093/nar/gku1258)
Supplement: SUPPLEMENTARY DATA [file supp_gku1258_Schmidt_2014_Supplemental_gku1258.pdf]

Supplementary Data

Identification of SMG6 cleavage sites and a preferred RNA cleavage motif by global analysis of endogenous NMD targets in human cells

Skye A. Schmidt, Patricia L. Foley, Dong-Hoon Jeong, Linda A. Rymarquis, Francis Doyle, Scott A. Tenenbaum, Joel G. Belasco, Pamela J. Green[1*]

Figure S1

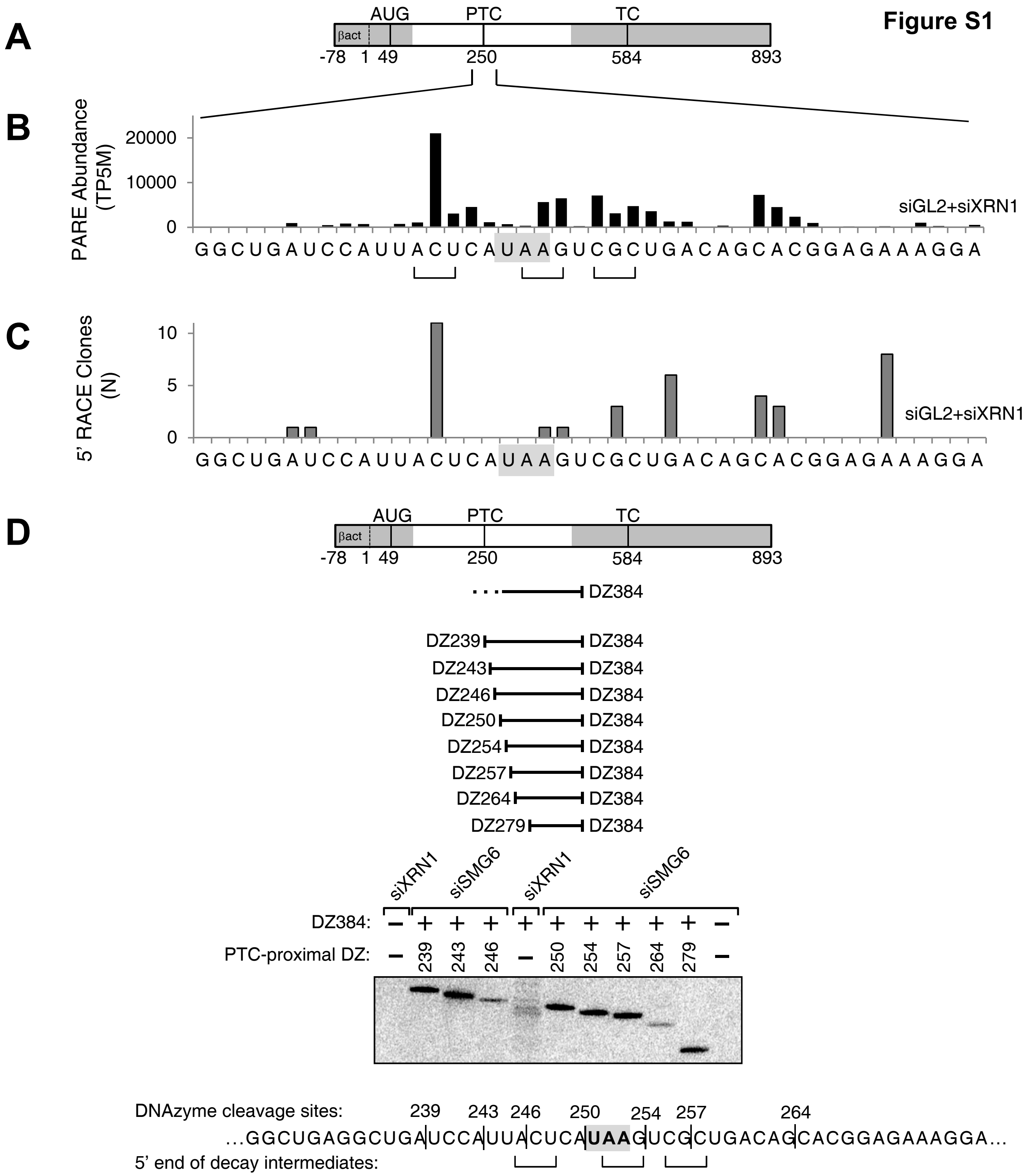

Figure S1. 5' ends of TCRβ68 cleavage products as determined by independent methods. A. Schematic diagram of the TCRβ68 reporter. B. Normalized abundance of PARE 5' ends from siGL2+siXRN1 libraries. The premature termination codon (PTC) is highlighted in gray. Brackets indicate the approximate location of cleavage sites mapped by DNAzyme analysis. C. 5' ends of 3'-terminal TCRβ68 decay intermediates detected by RLM-5' RACE. D. Northern blot mapping 5' ends by site-specific DNAzyme cleavage. Prior to electrophoresis, RNA from siXRN1-treated cells was treated with a DNAzyme (DZ384) to induce site-specific cleavage 133 nt downstream of the TCRβ68 PTC. In addition, size markers were generated by treating RNA from siSMG6-treated cells with DZ384 and any of several DNAzymes targeting sites surrounding the PTC. Above the blot is a map of the siSMG6 standards with respect to the TCRβ68 transcript. In the sequence below the blot, the vertical lines mark DNAzyme cleavage sites, and the bracketed nucleotides indicate the approximate location of the TCRβ68 5' ends identified by comparison to the DNAzyme-cleaved markers.

Figure S2

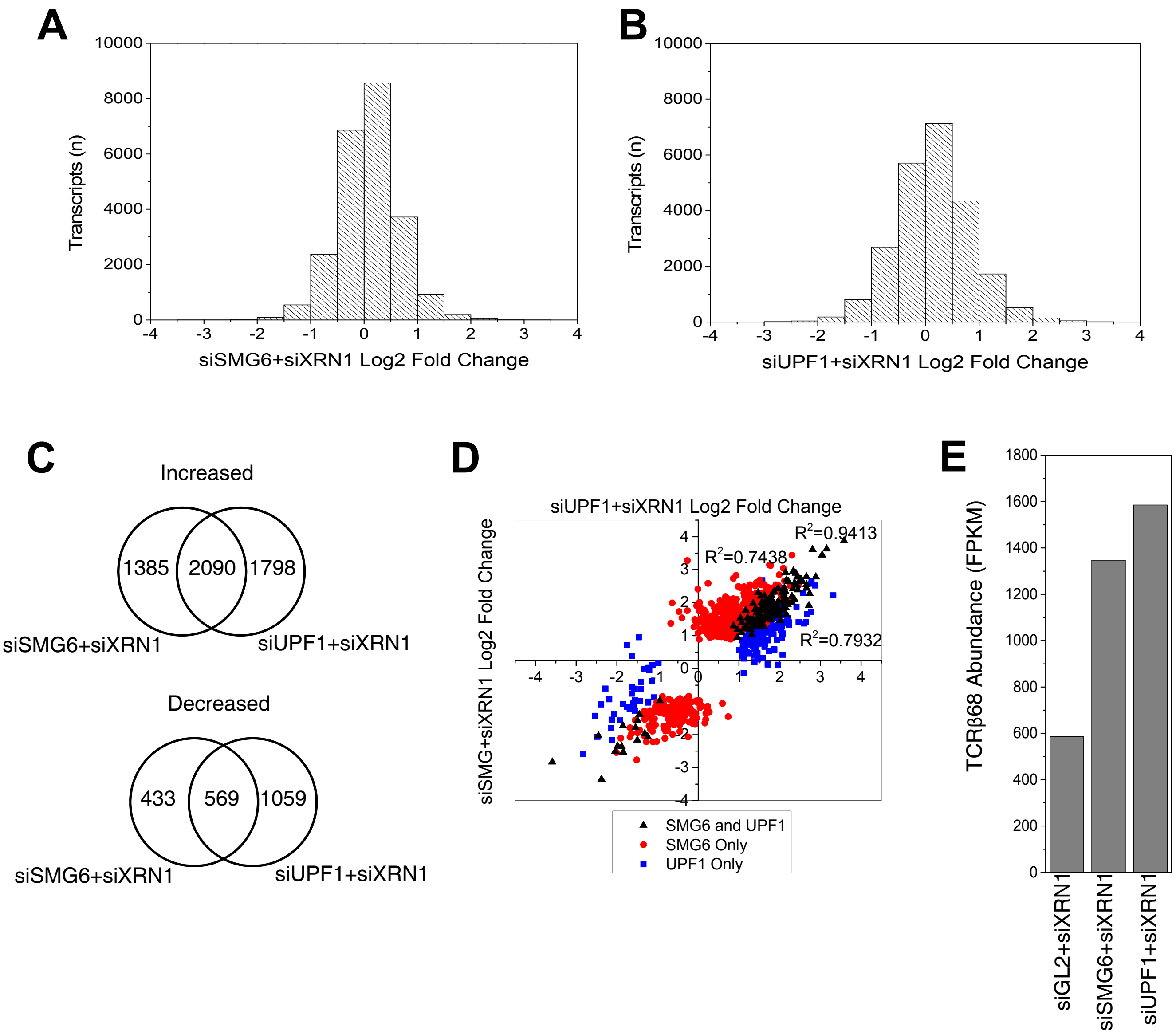

Figure S2. **Effect of SMG6 and UPF1 knockdown on overall mRNA abundance.** Fold change in the abundance of protein coding transcripts determined by RNA-Seq (n=34,085) following A. SMG6 knockdown or B. UPF1 knockdown. C. Number of protein coding transcripts whose abundance significantly increased or decreased upon SMG6 or UPF1 knockdown (CuffDiff, $q \leq 0.05$). D. Relationship between the change in transcript abundance resulting from SMG6 knockdown or UPF1 knockdown for transcripts significantly up- or down-regulated by knocking down both SMG6 and UPF1, SMG6 only, or UPF1 only. E. Abundance of the TCRβ68 reporter in the siGL2+siXRN1, siSMG6+siXRN1, and siUPF1+siXRN1 libraries.

Figure S3

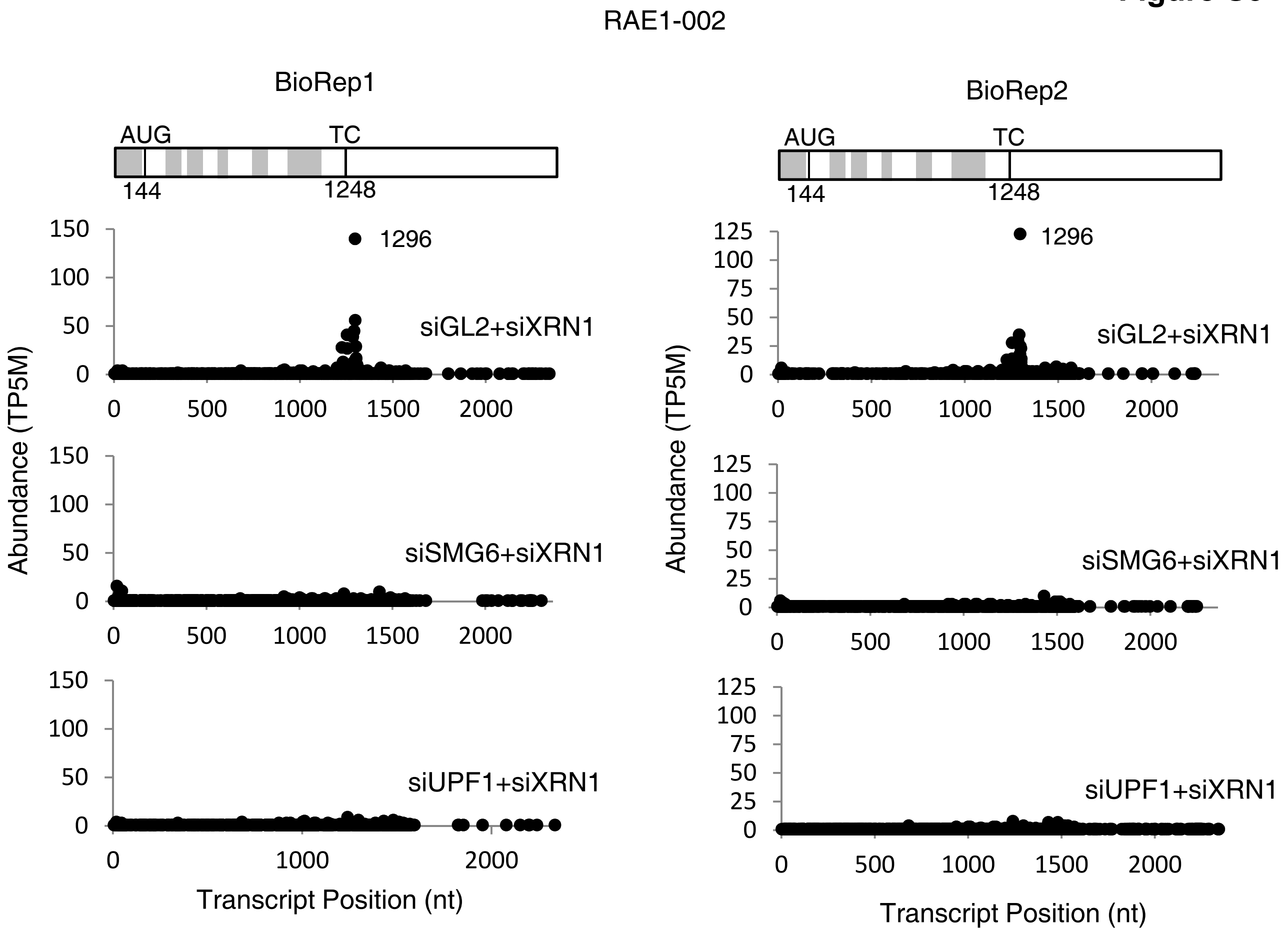

Figure S3. Reproducibility of D-plots for an endogenous transcript (RAE1-002) in different biological replicates. Left, Biorep 1. Right, Biorep 2. Alternating gray and white zones represent exons. AUG, translation initiation codon; TC, natural termination codon.

Figure S4

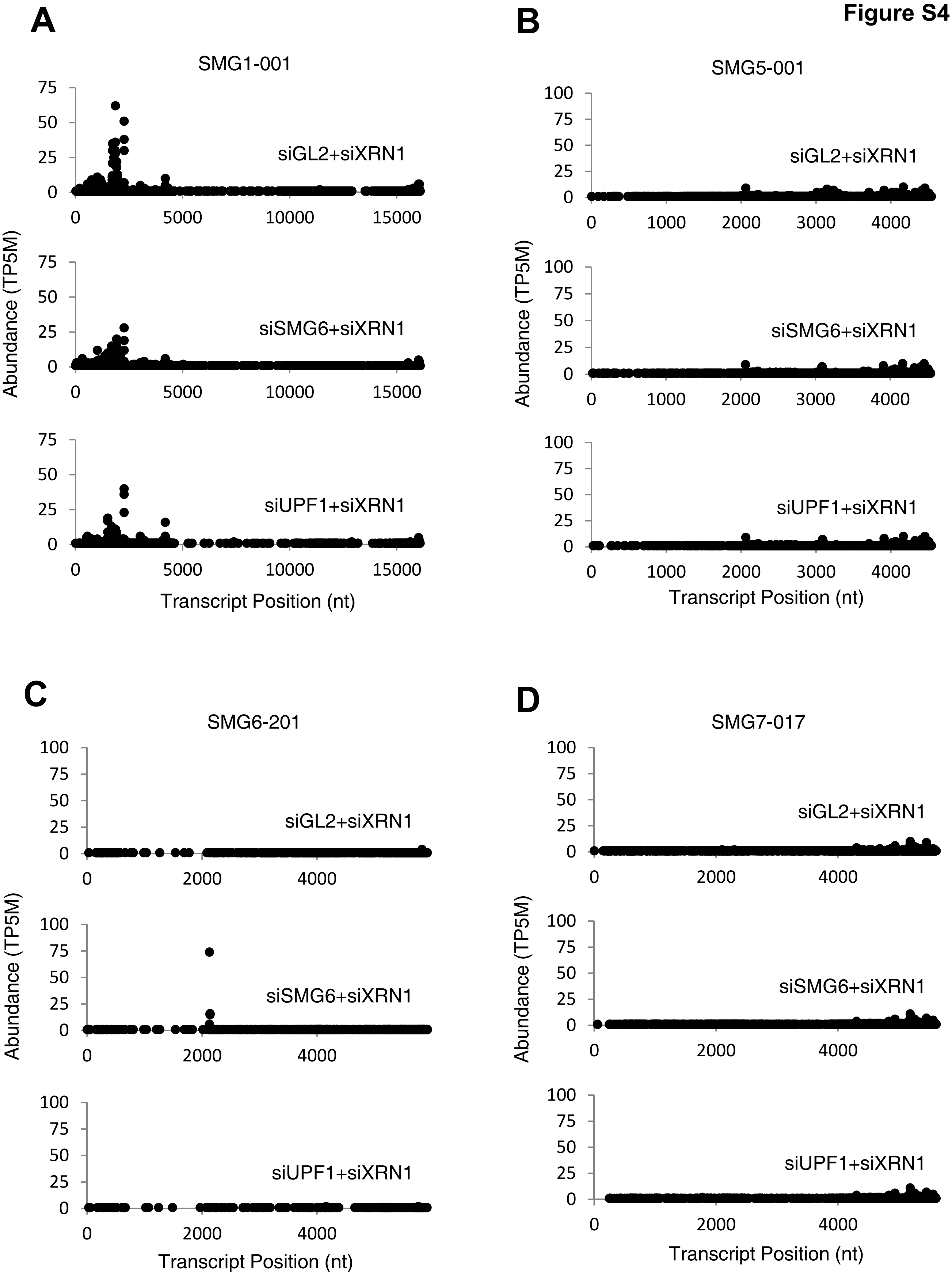

A
SMG1-001
siGL2+siXRN1
siSMG6+siXRN1
siUPF1+siXRN1
Abundance (TP5M)
Transcript Position (nt)
B
SMG5-001
siGL2+siXRN1
siSMG6+siXRN1
siUPF1+siXRN1
Abundance (TP5M)
Transcript Position (nt)
C
SMG6-201
siGL2+siXRN1
siSMG6+siXRN1
siUPF1+siXRN1
Abundance (TP5M)
Transcript Position (nt)
D
SMG7-017
siGL2+siXRN1
siSMG6+siXRN1
siUPF1+siXRN1
Abundance (TP5M)
Transcript Position (nt)

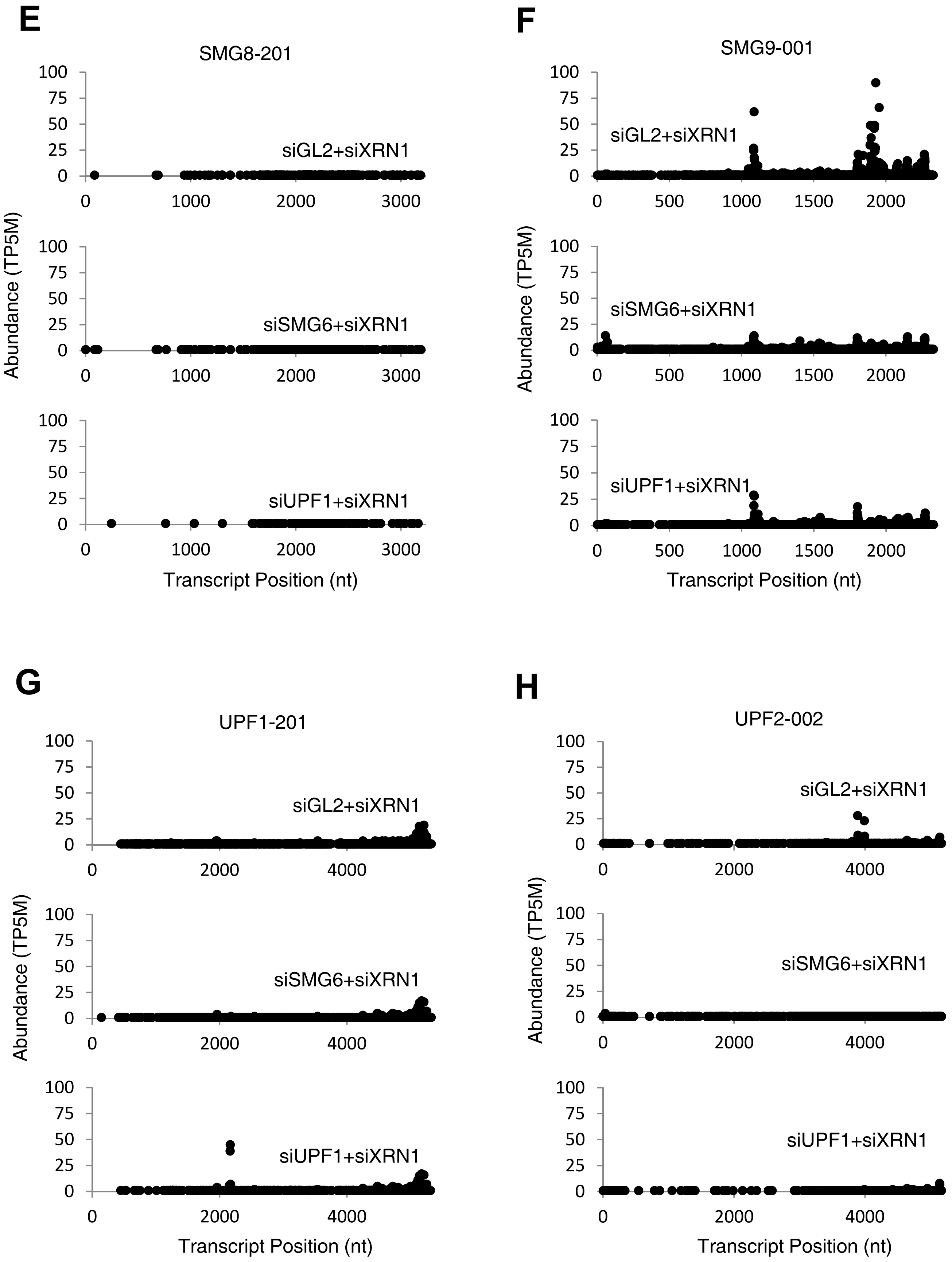
E
SMG8-201
siGL2+siXRN1
siSMG6+siXRN1
siUPF1+siXRN1
Abundance (TP5M)
Transcript Position (nt)
F
SMG9-001
siGL2+siXRN1
siSMG6+siXRN1
siUPF1+siXRN1
Abundance (TP5M)
Transcript Position (nt)
G
UPF1-201
siGL2+siXRN1
siSMG6+siXRN1
siUPF1+siXRN1
Abundance (TP5M)
Transcript Position (nt)
H
UPF2-002
siGL2+siXRN1
siSMG6+siXRN1
siUPF1+siXRN1
Abundance (TP5M)
Transcript Position (nt)

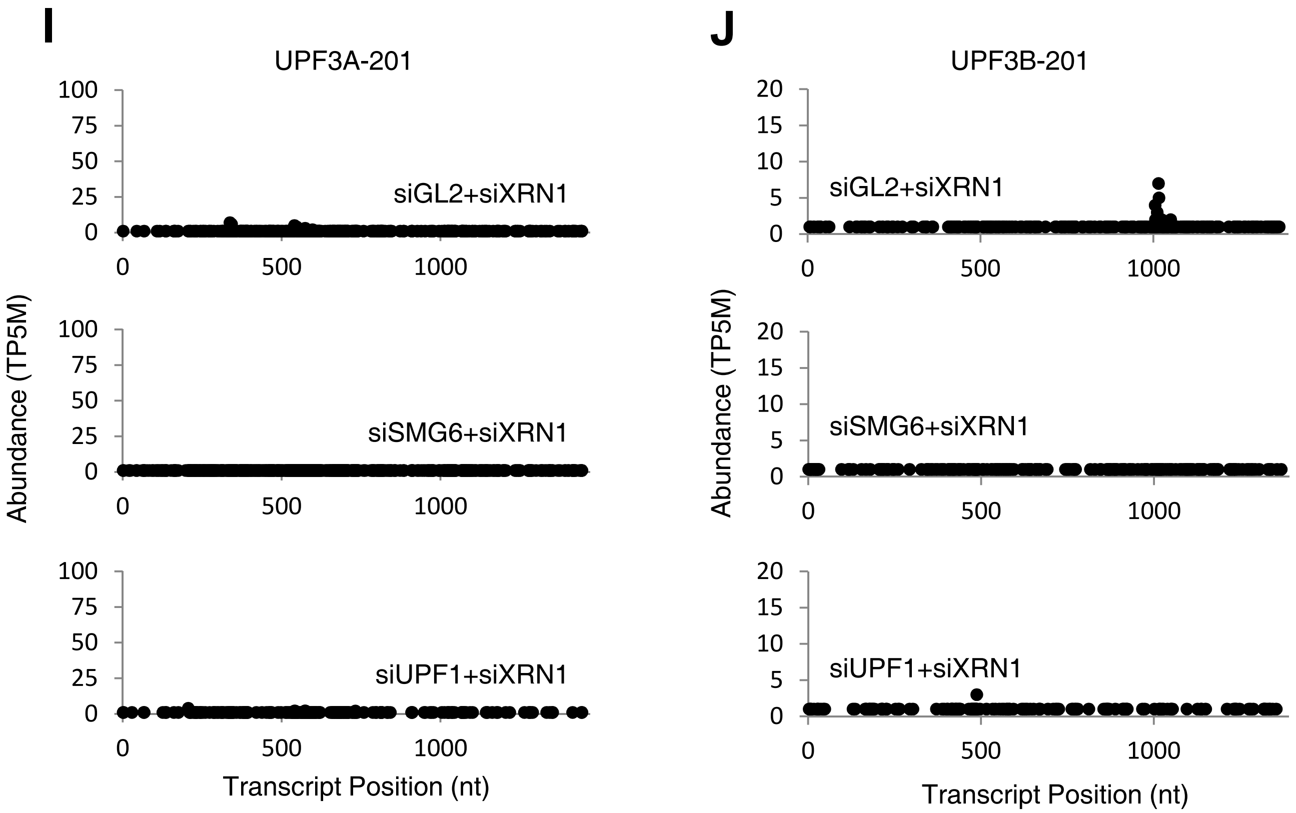

Figure S4. D-plots of transcripts encoding proteins involved in NMD. A. D-plot for SMG1-001, prominent MaxSeq at 1864 nt did not pass the MaxPeak Percent criteria. Transcripts were chosen based on representative transcript criteria. B. D-plot for SMG5-001, no prominent MaxSeq in any SMG5 transcript. C. D-plot for SMG6-001, no prominent MaxSeq in siGL2+siXRN1. The prominent peak in siSMG6+siXRN1 contains two prominent sequences corresponding to the siRNA-mediated cleavage of the transcript, as well as the siRNA. D. D-plot for SMG7-017; no prominent MaxSeq in any SMG7 transcript. E. D-plot for SMG8-201, no prominent MaxSeq in any SMG8 transcript. F. D-plot for SMG9-001, prominent MaxSeq at 1930 nt did not pass MaxPeak Percent criteria. G. D-plot for UPF1-201, no prominent MaxSeq in siGL2+siXRN1. The prominent peak in siUPF1+siXRN1 corresponds to the siRNA-mediated cleavage of the transcript, as well as the siRNA. H. D-plot for UPF2-002, two prominent MaxSeqs at positions 3888 nt in Biorep1, and 3988 nt in Biorep2. I. D-plot for UPF3A-001, MaxSeq at position 337 nt did not pass MaxSeq Abundance, MaxPeak Abundance, or MaxPeak Percent critiera. J. D-plot for UPF3B-201, identified as a SMG6 target. Although transcript variant UPF3B-201 was the only one that came through the pipeline, the PARE MaxSeq is located 28 nt upstream from the TC in UPF3B-001 and is the likely the variant triggering NMD.

Figure S5

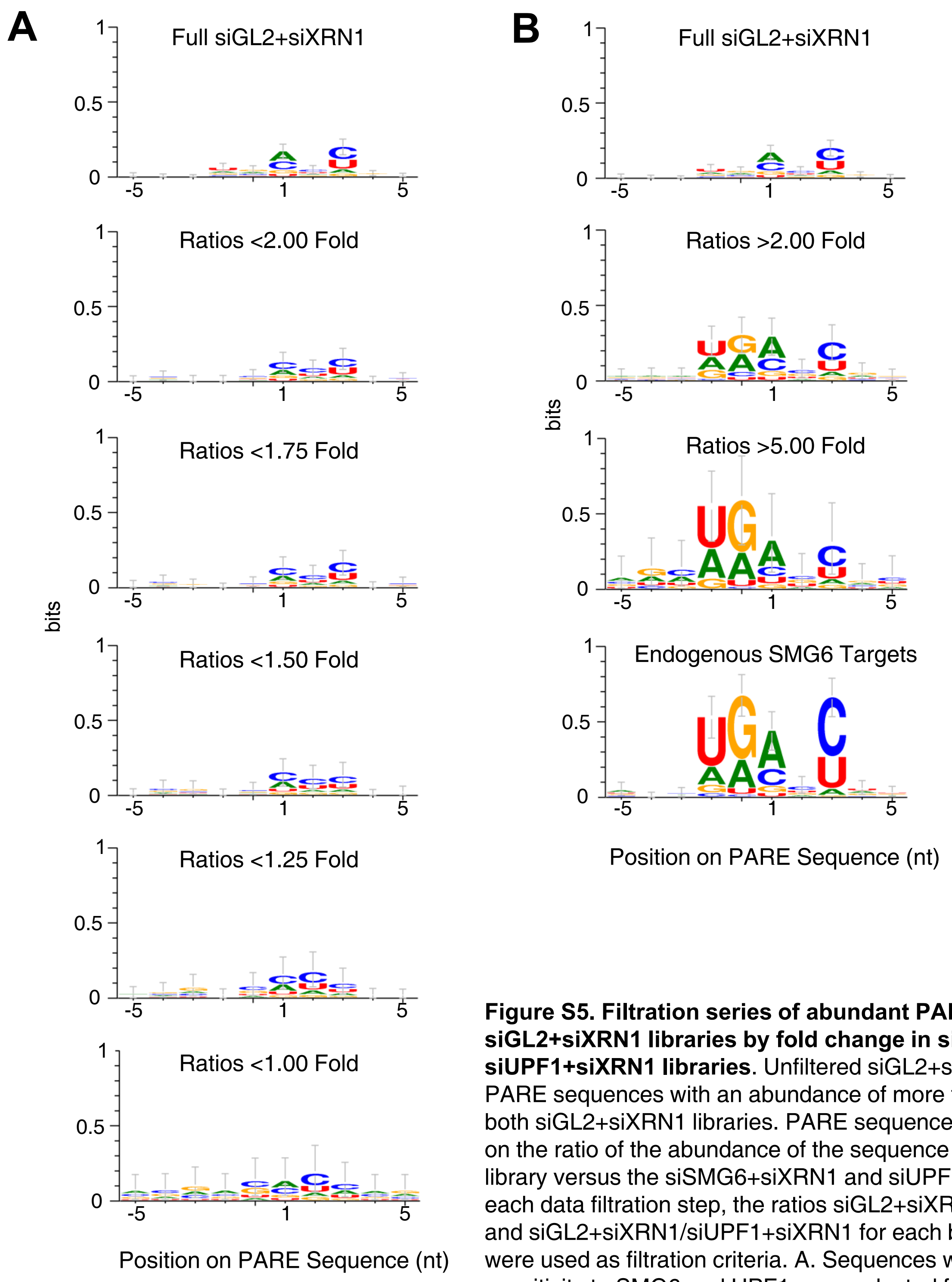

Figure S5. Filtration series of abundant PARE sequences in siGL2+siXRN1 libraries by fold change in siSMG6+siXRN1 and siUPF1+siXRN1 libraries. Unfiltered siGL2+siXRN1 represents PARE sequences with an abundance of more than 100 TP5M in both siGL2+siXRN1 libraries. PARE sequences were selected based on the ratio of the abundance of the sequence in the siGL2+siXRN1 library versus the siSMG6+siXRN1 and siUPF1+siXRN1 libraries. In each data filtration step, the ratios siGL2+siXRN1/siSMG6+siXRN1 and siGL2+siXRN1/siUPF1+siXRN1 for each biological replicate were used as filtration criteria. A. Sequences with decreasing sensitivity to SMG6 and UPF1 were selected for analysis in each filtration step. Unfiltered siGL2+siXRN1, n=677 nonredundant PARE sequences. Ratios < 2.00 fold, n=288. Ratios < 1.75 fold, n=238. Ratios < 1.50 fold, n=177. Ratios < 1.25 fold, n=118. Ratios < 1.00 fold, n=68. B. Sequences with increasing sensitivity to SMG6 and UPF1 were selected for analysis in each filtration step. Ratios > 2.00 fold, n=179. Ratios > 5.00 fold, n=40.

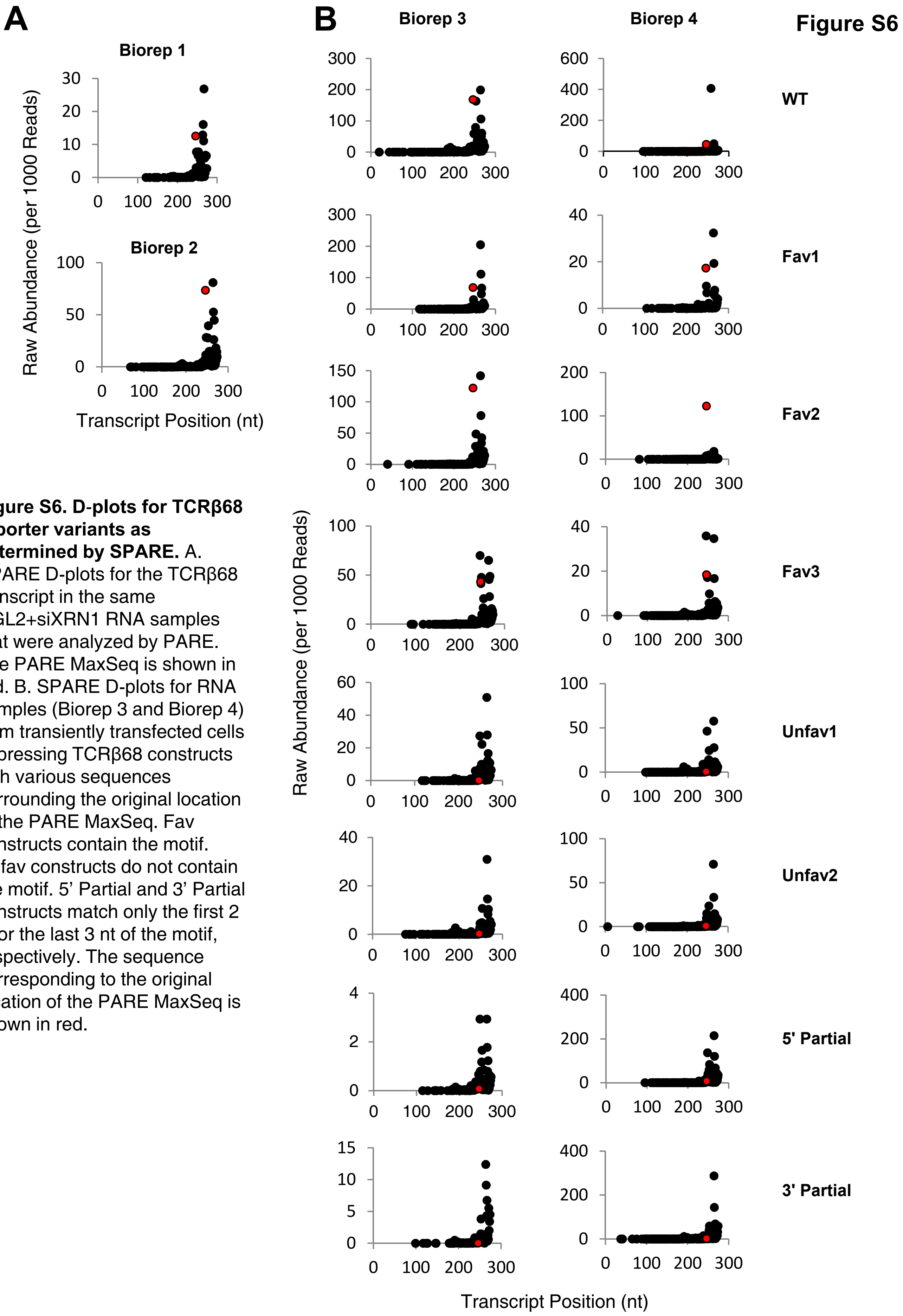

Figure S6. D-plots for TCRβ68 reporter variants as determined by SPARE. A. SPARE D-plots for the TCRβ68 transcript in the same siGL2+siXRN1 RNA samples that were analyzed by PARE. The PARE MaxSeq is shown in red. B. SPARE D-plots for RNA samples (Biorep 3 and Biorep 4) from transiently transfected cells expressing TCRβ68 constructs with various sequences surrounding the original location of the PARE MaxSeq. Fav constructs contain the motif. Unfav constructs do not contain the motif. 5’ Partial and 3’ Partial constructs match only the first 2 nt or the last 3 nt of the motif, respectively. The sequence corresponding to the original location of the PARE MaxSeq is shown in red.

Figure S7

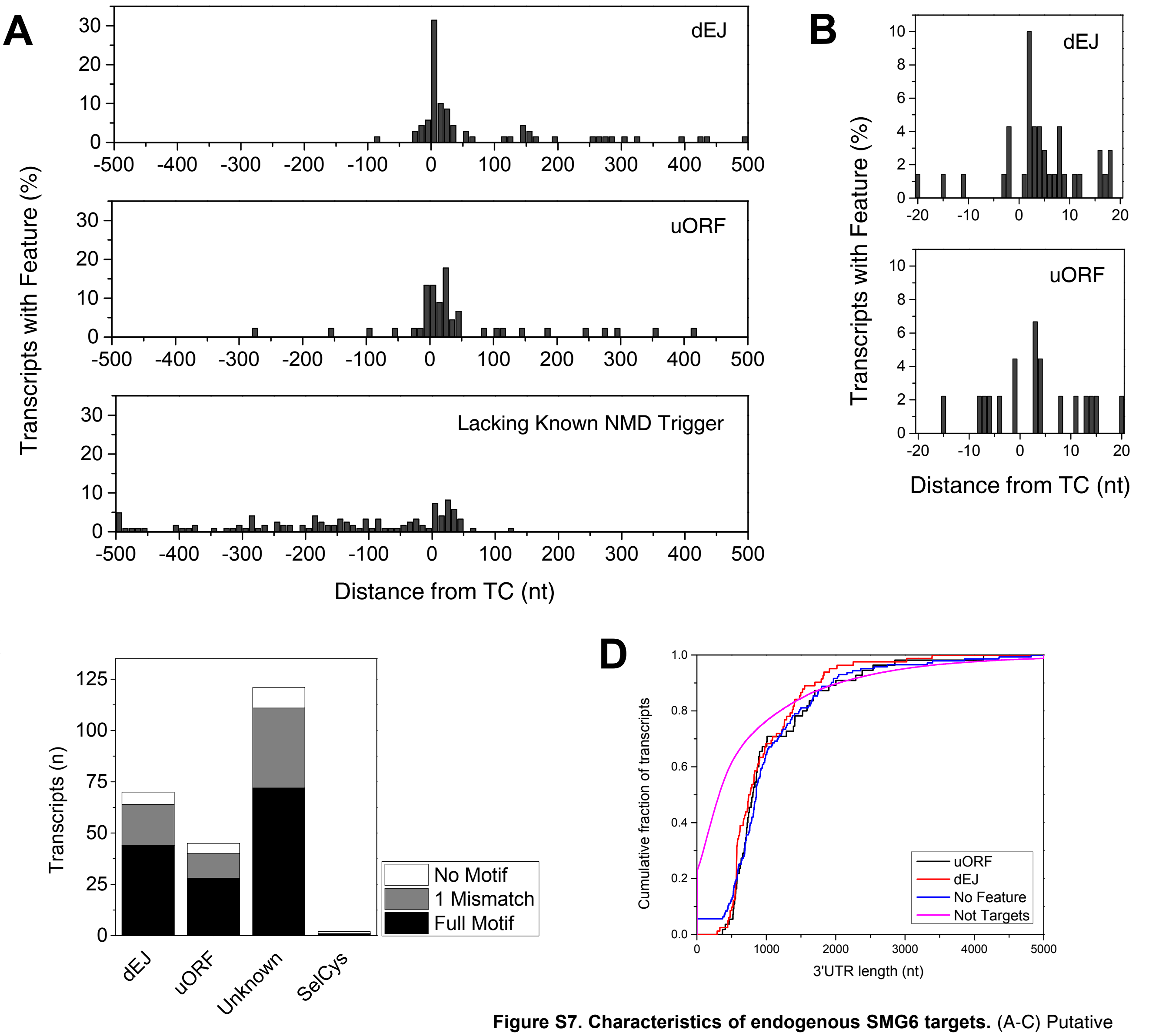

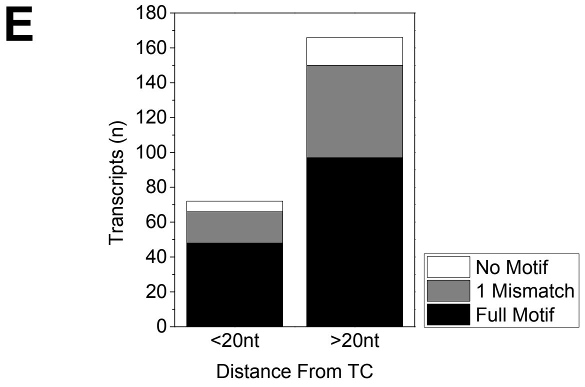

Figure S7. Characteristics of endogenous SMG6 targets. (A-C) Putative NMD-triggering features. SMG6 targets were evaluated for the presence of a known NMD-triggering feature, such as an upstream open reading frame (uORF), an exon junction more than 50 nt downstream of the termination codon (dEJ), or a selenocysteine UGA codon (SelCys). Among all transcripts containing a single PARE site, the transcript containing a canonical NMD-triggering feature, the highest mRNA abundance, or the closest distance of the PARE MaxSeq to a termination codon was chosen as a representative transcript for that PARE site. If a transcript lacked a dEJ, uORF, and SelCys, it was classified as having no known feature. A. Distance from the SMG6-dependent PARE site to the nearest termination codon for transcripts with various potential NMD-inducing features, binned in 10 nt intervals. B. Distance from the PARE site to the termination codon for transcripts with various potential NMD-inducing features, at single nt resolution. C. Occurrence of complete or 1 nt-mismatched SMG6 motif in representative transcripts with various potential NMD-inducing features. D. Length of the 3' UTR of endogenous SMG6 targets**.** Cumulative data function showing the length of the 3' UTR for transcripts that were identified by PARE as SMG6 targets (uORF, dEJ, No feature) as compared to all other protein-coding transcripts in Gencode V11 (Not targets). SMG6 targets were divided into categories based on whether an identifiable NMD triggering feature was annotated. uORF, upstream open reading frame, n = 45; dEJ, exon junction more than 50 nt downstream of the termination codon, n= 70; No feature, no identifiable NMD trigger, n =121; Not targets, n = 91,525. E. Presence of a full or 1 nt-mismatched motif in proximity to a termination codon in representative transcripts.

Table S1. A. Summary statistics for PARE libraries.

Library	Type[1]	Description	Trimmed Abundance[2]	Trimmed Distinct[3]	Genome-Matched Abundance[4]	Genome-Matched Distinct[5]
HSA269	monoP	TCR beta68; siGL2+siXRN1; Bio1	86,038,213	10,004,189	32,749,466	8,794,261
HSA270	monoP	TCR beta68; siSMG6+siXRN1; Bio1	98,920,855	8,687,180	25,712,440	7,267,643
HSA271	monoP	TCR beta68; siUPF1+siXRN1; Bio1	90,932,287	4,347,557	10,018,893	3,726,048
HSA359	monoP	TCR beta68; siGL2+siXRN1; Bio2	15,235,222	3,630,297	8,793,889	3,399,989
HSA360	monoP	TCR beta68; siSMG6+siXRN1; Bio2	99,230,454	10,354,486	43,213,331	9,683,048
HSA361	monoP	TCR beta68; siUPF1+siXRN1; Bio2	193,011,070	16,382,581	76,742,286	14,843,903
HSA276	Cap	TCR beta68; siGL2+siXRN1; Bio1	27,478,434	1,484,735	25,348,347	730,654
HSA277	Cap	TCR beta68; siSMG6+siXRN1; Bio1	38,566,652	2,465,866	35,132,916	1,235,824
HSA278	Cap	TCR beta68; siUPF1+siXRN1; Bio1	34,688,235	1,918,198	31,979,899	975,061
HSA387	Cap	TCR beta68; siGL2+siXRN1; Bio2	21,535,845	724,893	20,131,835	602,739
HSA388	Cap	TCR beta68; siSMG6+siXRN1; Bio2	18,704,808	851,227	17,218,508	740,927
HSA389	Cap	TCR beta68; siUPF1+siXRN1; Bio2	20,540,845	692,136	19,360,653	575,242

B. Summary Statistics for RNA-Seq libraries**.**

Library	Description	Trimmed Read Length[6]	Trimmed Abundance[2]	Trimmed Distinct[3]	Genome Matched Abundance[4]	Genome Matched Distinct[5]
HSA609	TCR beta68; siGL2+siXRN1; Bio1	49 nt	25,357,728	10,852,701	23,379,219	10,099,177
HSA610	TCR beta68; siSMG6+siXRN1; Bio1	49 nt	19,230,809	9,113,399	17,817,508	8,561,044
HSA611	TCR beta68; siUPF1+siXRN1; Bio1	49 nt	32,550,547	13,840,890	29,962,427	12,742,148
HSA612	TCR beta68; siGL2+siXRN1; Bio2	49 nt	32,991,362	15,966,397	29,232,091	14,782,076
HSA613	TCR beta68; siSMG6+siXRN1; Bio2	49 nt	19,954,265	12,258,162	17,844,255	11,369,111
HSA614	TCR beta68; siUPF1+siXRN1; Bio2	49 nt	28,351,211	16,265,099	25,252,280	14,935,656

[1]The type of PARE Library constructed. monoP refers to a regular PARE library, while Cap refers to a PARE library constructed from capped RNA
[2]The total number of sequences obtained for the library
[3]The total number of nonredundant trimmed sequences
[4]The number of sequences that match human genome hg19
[5]The number of nonredundant sequences that match human genome hg19 and *TCRβ68* transgene
[6]Reads trimmed based on quality scores as described in Methods

Table S2. GO categories that are over-represented in the SMG6 target set compared to all expressed genes.

GO Term	Fold Enrichment	p-value
Response to reactive oxygen species	7.4	0.00031
Cellular response to stress	2.3	0.00076
Response to DNA damage stimulus	2.4	0.00203
Cell death	2.0	0.00379
Death	2.0	0.00404
Response to hydrogen peroxide	7.3	0.00453
Response to oxidative stress	3.7	0.00552
Region of interest:DNA binding	23.0	0.00661
Apoptosis	2.1	0.00765
CDP-alcohol phosphatidyltransferase activity	20.9	0.00824
Programmed cell death	2.0	0.00872

EXTENDED MATERIALS AND METHODS

Plasmids and cell lines

HeLa cell lines that stably express TCRβwt (pβ433) and TCRβ68 (pβ434) reporter transcripts, and plasmids that express wildtype SMG6 (pSmg6^R) and a catalytically-inactive SMG6 mutant (pSmg6^R-m1, referred to as Smg6^R-mut in the text and in Figure 1) were described previously (1,2). For SMG6 motif analysis, plasmid pTCRβ68U was generated by inserting the non-human portion of the TCRβ68 gene into the plasmid pCL-6E1 (3) between the SacI and XbaI sites. Mutations were made to the SMG6 motif using overlapping PCR with the primers described below.

RNAi-mediated knockdowns and RNA isolations

Cells were seeded at a density of 2.5x10^4 cells/cm^2, and grown in DMEM supplemented with 10% FBS and 2 mM L-glutamine. After 24 hrs, cells were co-transfected with siXRN1 (20 nM), and siGL2 (control), siSMG6, or siUPF1 (20 nM). After 48 hrs, the medium was exchanged for fresh medium and cells were re-transfected. After another 24 hrs, the cell layers were washed 3 times with ice-cold PBS and RNA was extracted in Trizol LS reagent (Life Technologies) according to the manufacturer's recommendations. Lipofectamine 2000 (Life Technologies) was used for all transfections according to the manufacturer's recommendations. For SMG6 complementation experiments and TCRβ68 motif analysis, cells were grown in 10 cm dishes, and were transfected with 4 µg plasmid DNA 48 hrs after seeding. Knockdown efficiencies were verified by immunoblot analysis (XRN1, UPF1) or by RT-qPCR (SMG6).

Immunoblot analysis

Crude protein extracts of transfected cells were fractionated by electrophoresis on 8% polyacrylamide-SDS gels and electrotransferred to nitrocellulose. After blocking with 3% dry milk in phosphate-buffered saline containing 0.05% Tween-20 (PBS-T), the blots were probed for 2 hr at room temperature with rabbit monoclonal anti-XRN1 antibodies (diluted 1:4,000 in PBS-T containing 0.3% dry milk, Bethyl Labs), rabbit monoclonal anti-RENT1 antibodies (1:3,000, Bethyl Labs), or rabbit monoclonal anti-GAPDH antibodies (1:3,000, Rockland). Then blots were incubated with a secondary antibody conjugated to IR700 dye (1:20,000 in PBS-T containing 0.3% dry milk, Rockland), and detected with the Odyssey Infrared Imaging System (Licor). Band intensities were quantified using Odyssey application software version 2.1.

Quantitative reverse transcription-PCR (qRT-PCR)

cDNA was synthesized from 1 μg of total cellular RNA by using SuperScript III (Invitrogen), along with an oligo$(dT)_{20}$ primer (Invitrogen), according to the manufacturer's recommendations. qPCR was carried out in triplicate in a volume of 30 μL, which included 15 μL of SYBR green supermix (Roche). After heating for 10 min at 95°C, the qPCR mixtures were subjected to 40 amplification cycles (15 s at 95°C, 1 min at 55°C). The initial concentration of each cDNA was determined by fitting the data to a two-parameter MAK2 model (4,5), using an offset of four cycles after the second derivative maximum to approximate the first derivative maximum, and then normalized to that of the GAPDH mRNA.

Northern blot analysis

For Figure 1, equal amounts of total cellular RNA (10 µg) were denatured, fractionated by electrophoresis on a 1.2% formaldehyde agarose gel, transferred by capillary action to a Immobilon-NY+ nylon membrane (Millipore) and crosslinked. For Figure S1, samples were separated on an 8% polyacrylamide-8 M urea denaturing gel, electroblotted and cross-linked onto an Immobilon-NY+ nylon membrane (Millipore). Blots were probed at 65°C in PerfectHyb Plus hybridization buffer (Sigma) with internally radiolabeled DNA probes generated using High Prime (Roche), and then washed with 2× SSC (1× SSC is 0.15 M NaCl plus 0.015 M sodium citrate)-0.1% sodium dodecyl sulfate (SDS) buffer at room temperature, then 0.1x SSC-0.1% SDS at 65°C. The probe template was amplified from HeLa TCRβ68 cDNA with primers TCRβ68 V+ and TCRβ68 C-, and corresponded to codons 60 - 101. Northern blot band intensities were quantified by using a Molecular Dynamics Storm 820 Phosphorimager.

Validation of SMG6 cleavage sites in TCRβ68 with DNAzymes

Total cellular RNA (10 µg) collected from HeLa TCRβ68 cells that were treated with siXRN1 were combined with 0.4 pmol DNAzyme DZ384. To serve as standards, aliquots of total cellular RNA (10 µg) collected from HeLa TCRβ68 cells treated with siSMG6 were combined with 0.4 pmol DNAzyme DZ384 and 0.4 pmol of one of several DNAzymes designed to cleave in the vicinity of the PTC (See table below). Samples were heated to 75°C for 5 min, and then cooled slowly to 30°C. To each reaction, 10x DZ buffer (50 mM Tris-HCl pH 7.5, 10 mM $MgCl_2$, 1 mM ATP, 10 mM DTT) was added to a final concentration of 1x, and the reactions were incubated at 37ºC for 4 hr. Cleaved RNA fragments (10 µg total RNA for the siXRN1 sample, 2 µg total RNA for the siSMG6 samples) were analyzed by northern blot.

Validation of SMG6 cleavage in TCRβ68 with RLM-RACE

A modified procedure for RNA ligase-mediated (RLM) 5' rapid amplification of cDNA ends (5' RACE) was performed using the FirstChoice RLM-RACE kit (Ambion) as described (6). Two μg of total RNA from HeLa TCRβ68 cells that had been treated with siGL2+siXRN1 were ligated to a 5' RACE RNA adaptor without calf intestine alkaline phosphatase treatment. cDNA was synthesized using a gene-specific outer primer, then initial PCR was performed using the 5'RACE outer primer and a gene-specific outer primer. Nested PCR was carried out using 1/50 of the initial PCR, the 5' RACE inner primer, and a gene-specific inner primer. RACE fragments (~ 250 nt) were cloned into the pGEM T-easy vector after gel purification (Invitrogen) and sequenced.

PARE library construction

PARE libraries were constructed from poly(A)$^+$ mRNA isolated from 300 μg of total RNA starting material using Absolute mRNA purification kit (Agilent Technologies). PARE libraries were constructed as previously described (7,8) as modified for Illumina HiSeq platform (9). Libraries were individually sequenced on the Illumina HiSeq 2000 platform.

Computational analysis of PARE libraries

PARE bioinformatic analysis was done using custom Perl scripts. After removing adapter sequences, raw reads were trimmed to 20 nt. Trimmed PARE data was perfectly matched to TCRβ68 using Bowtie 0.7.12 (10). Sequences were then matched to the human genome hg19 (http://hgdownload.cse.ucsc.edu/downloads.html) and Gencode V11 protein-coding sequences (http://www.gencodegenes.org/releases/11.html) with 1 nt reiterative mismatching. Total

sequences were first matched to an index allowing 0 mismatches, then remaining unmatched sequences were rematched to the same index allowing 1 mismatch. Sequences that matched the human genome more than 100 times, as well as sequences that matched to the mitochondrial genome, and repeat-containing sequences defined as sequences containing a continuous 12 nt of single, double, or triple nucleotide repeats, were removed. Abundance was normalized to transcripts per 5 million genome-matched reads.

Direct targets of SMG6 cleavage were identified using the custom Perl scripts. Total PARE abundance was calculated as the sum of abundance of all PARE sequences that match a transcript. The most abundant PARE sequence matching an individual transcript was identified as the MaxSeq. MaxSeq Abundance was defined as the sum of all sequences starting at the same 5' position as the MaxSeq. The PARE sequences in a window extending ± 10 nt from the MaxSeq were together defined as the MaxPeak, and the abundance of the MaxPeak was the sum of the abundance of all sequences in that window. The parameter MaxPeak Percent was the proportion of total abundance of all PARE sequences matching the transcript for which the MaxPeak accounted, and was calculated as MaxPeak Abundance/Total Abundance *100. Cutoffs for MaxSeq and MaxPeak abundance were chosen to select the upper 25% of all 6 libraries, and the MaxPeak Percent cutoff was chosen to select the upper 50% of all libraries. In many genes, the same MaxSeq was detected for several splice variants. In some cases, the MaxSeq differed between splice variants. Therefore to reduce redundancy, a PARE site was defined as a distinct PARE sequence within a gene.

In the pipeline in Figure 3, the genome-matched PARE sequences were first filtered for those that matched protein-coding sequences. In the second filter, the MaxSeqs in siGL2+siXRN1 were identified that met a set of Prominence Criteria which required a MaxSeq in the same position in both biological replicates, MaxSeq Abundance ≥ 8 TP5M, a MaxPeak Abundance ≥ 25 TP5M, and a MaxPeak Percent ≥ 15% in both biological replicates. For the third filter step, Abundance Ratio Criteria, the MaxSeqs previously identified needed to be at least 1.5-fold higher in the siGL2+siXRN1 libraries than either siSMG6+siXRN1 or siUPF1+siXRN1 in individual biological replicates, and an average of 2.0 times higher when the biological replicates are averaged. The Transcript Abundance Critieria filter then selected for transcripts whose abundance in siSMG6+siXRN1 libraries was ≥ 1.00 FPKM and not significantly higher in siGL2+siXRN1 libraries than in siSMG6+siXRN1 or siUPF1+siXRN1. The final filter step of Redundancy Minimization required that the number of MaxSeq Genome hits (matches) be ≤ 2 and that the MaxSeq not match more than one target gene.

C-PARE library construction

C-PARE libraries were constructed from poly(A)$^+$ mRNA isolated from 300 μg of total RNA as starting material using the Absolute mRNA purification kit (Agilent Technologies). Poly(A)$^+$ mRNA was dephosphorylated with calf intestinal alkaline phosphatase (Invitrogen) treatment at 37°C for 1 hr. After terminating the reaction with phenol/chloroform extraction and ethanol precipitation, mRNA was decapped using tobacco acid pyrophosphatase (Epicenter) at 37°C for 1 hr and cleaned with phenol/chloroform extraction and ethanol precipitation. After these pretreatments, the mRNA was used for PARE library construction as previously described (9). Four libraries were pooled and sequenced in a channel on the Illumina HiSeq 2000 platform.

Computational Analysis of C-PARE Libraries

After removing adapter sequences, raw reads were trimmed to 20 nt. Trimmed PARE data was matched to hg19 as in PARE libraries, in addition to a custom index comprised of Gencode V11 protein-coding transcripts concatenated with 100 nt upstream sequence from annotated TSS extracted from the genome. Abundance was normalized to transcripts per 10 million reads.

SPARE for TCRβ68 motif analysis

Poly(A)$^+$ mRNA was isolated from 10 μg of total RNA using Absolute mRNA purification kit (Agilent Technologies). mRNA was denatured at 65°C for 5 min and ligated to a 5' RNA adaptor using T4 RNA ligase at 37°C for 1 hr. After cleaning the reaction by acid phenol:chloroform extraction and ethanol precipitation, the first strand of cDNA was synthesized by using a TCRβ68 specific RT primer and Superscript III reverse transcriptase (Invitrogen) at 42°C for 1 hr. After phenol:chloroform extraction and ethanol precipitation, the cDNA was dissolved in 5 μl of water. A PCR was carried out using the SPARE Fw and Rv primers for 24 cycles. The primers were removed from the reactions with Agencourt AMPure XP Beads (Beckman Coulter) and a second round of PCR was carried out using the P5 primer and the P7 index primer for 21 cycles. Final PCR products were separated in a 6% acrylamide gel and gel bands ranging in size from 150 to 225 nt were excised and purified. Equal amounts of cDNA from all of the libraries were pooled and sequenced (18/channel) in an Illumina HiSeq 2000 platform.

Computational analysis of SPARE libraries

Library quality was determined using FastQC (http://www.bioinformatics.babraham.ac.uk/projects/fastqc/). Libraries were trimmed of poor quality bases using FASTX-Toolkit (http://hannonlab.cshl.edu/fastx_toolkit/). Sequences that matched the 5' PARE adapter or the TCRβ68 RT primer were removed from SPARE libraries using CutAdapt (11). Sequences greater than 51 nt were discarded. Sequences were then exactly matched to the expected TCRβ68U sequence using Bowtie 0.7.12. The most abundant sequence in all 8 variants occurred at position 264. Sequences were internally normalized by dividing the sequence abundance by the abundance at position 264.

RNA-Seq library construction

Five µg of total RNA was enriched for poly(A)$^{+}$ mRNA using Absolute mRNA purification kit (Agilent Technologies). RNA-Seq libraries were constructed using TruSeq RNA sample prep kit (Illumina). Libraries were sequenced in pools of three per channel on a Illumina HiSeq 2000.

Computational analysis of RNA-Seq libraries

RNA-Seq library quality was determined using FastQC (http://www.bioinformatics.babraham.ac.uk/projects/fastqc/). Libraries were trimmed of poor quality bases using FASTX-Toolkit (http://hannonlab.cshl.edu/fastx_toolkit/). Sequences were matched to the custom hg19+TCRβ68 index using TopHat v2.0.4 (12) with Bowtie1, allowing for 1 mismatch and no novel junctions. Gencode V11 junction coordinates were obtained from the GTF file (http://www.gencodegenes.org/releases/11.html). Transcript abundance was determined using CufflinksV1.3.0 (http://cufflinks.cbcb.umd.edu/) and normalized to compatible hits. Differential analysis was done using the CuffDiff v2.0 program (13) using False Discovery

Rate-adjusted p-value of 0.05. Analysis was restricted to protein-coding transcripts with an average siSMG6+siXRN1 abundance ≥ 1.00 FPKM.

GO analysis of SMG6 targets

A background list of genes was generated by including all genes that were detected by the RNA-Seq analysis. Genes for which no RNA-Seq signal was detected in three or more of the biological samples were eliminated, leaving a total of 12,548 genes in the background list. Target and background gene lists were uploaded to the online DAVID bioinformatics analysis platform (http://david.abcc.ncifcrf.gov/home.jsp) (14). A functional annotation chart was generated using the default settings of the DAVID toolset.

SMG6 motif analysis

Sequence logos were created from the 10 nt surrounding the PARE MaxSeq using Weblogo 3 (15) with equiprobable nucleotide frequencies for the endogenous SMG6 targets and pipeline controls. Pipeline controls are a non-redundant set of sequences from transcripts that passed the Endonucleolytic Cleavage-Like Pipeline but whose MaxSeq abundance was less than 1.2-fold higher in siGL2+sXRN1 libraries than siSMG6+siXRN1, and siUPF1+siXRN1, and not located in the first or last 50 nt of the transcript. Abundant sequence controls were a non-redundant set of all PARE sequences not located in the first or last 50 nt of the transcript with an abundance higher than 100 TP5M in both of the siGL2+siXRN1 biological replicates. The expected frequency that a 5 nt sequence that matched the SMG6 motif at 1, 2, 3, or 4 positions would occur by chance was calculated by assuming the frequency of each nucleotide in the transcriptome was equal. Actual frequencies are (A = 0.26, T = 0.24, G = 0.25, C = 0.25).

Identification of potential NMD triggers

Upstream open reading frames (uORF) were predicted using EMBOSSv6.3.1 (http://emboss.sourceforge.net/) (16) requiring an AUG upstream of the annotated TSS, an in-frame stop codon upstream of, but not synonymous with, the stop codon for the main ORF, and a minimum length of 9 nt including the TC. To find downstream exon junctions (dEJs), exon junction positions for each transcript were identified from the Gencode V11 annotation and dEJs were defined as EJs $\geq$ 50 nt from the TC. Selenocysteine-containing transcripts were identified from the Gencode V16 annotation as that information was not available in V11.

Name	Sequence (5'-3')	Purpose
DNA Oligonucleotides		
TCRβ68 Fav1+	ATCCATAGACTATAAGTCGCTGACAGC	Primer to change SMG6 cleavage motif from UACUC to AGACU
TCRβ68 Fav1-	GACTTATAGTCTATGGATCAGCCTCAG	Primer to change SMG6 cleavage motif from UACUC to AGACU
TCRβ68 Fav2+	ATCCATTGCTTATAAGTCGCTGACAGC	Primer to change SMG6 cleavage motif from UACUC to UGCUU
TCRβ68 Fav2-	GACTTATAAGCAATGGATCAGCCTCAG	Primer to change SMG6 cleavage motif from UACUC to UGCUU
TCRβ68 Fav3+	ATCCATAAACCATAAGTCGCTGACAGC	Primer to change SMG6 cleavage motif from UACUC to AAACC
TCRβ68 Fav3-	GACTTATGGTTTATGGATCAGCCTCAG	Primer to change SMG6 cleavage motif from UACUC to AAACC
TCRβ68 Unfav1+	ATCCATCCTAGATAAGTCGCTGACAGC	Primer to change SMG6 cleavage motif from UACUC to CCUAG
TCRβ68 Unfav1-	GACTTATCTAGGATGGATCAGCCTCAG	Primer to change SMG6 cleavage motif from UACUC to CCUAG
TCRβ68 Unfav2+	ATCCATGTGGAATAAGTCGCTGACAGC	Primer to change SMG6 cleavage motif from UACUC to GUGGA
TCRβ68 Unfav2-	GACTTATTCCACATGGATCAGCCTCAG	Primer to change SMG6 cleavage motif from UACUC to GUGGA
TCRβ68 5'partial+	ATCCATAATAGATAAGTCGCTGACAGC	Primer to change SMG6 cleavage motif from UACUC to AAUAG
TCRβ68 5'partial-	GACTTATCTATTATGGATCAGCCTCAG	Primer to change SMG6 cleavage motif from UACUC to AAUAG
TCRβ68 3'partial+	ATCCATCCACCATAAGTCGCTGACAGC	Primer to change SMG6 cleavage motif from UACUC to CCACC
TCRβ68 3'partial-	GACTTATGGTGGATGGATCAGCCTCAG	Primer to change SMG6 cleavage motif from UACUC to CCACC
TCRβ-239	TGAGTAATGGAGGCTAGCTACAACGACAGCCTCAGC	10-23 DNAzyme to cleave TCRβ68 transcript 11 nt upstream of the PTC
TCRβ-243	CTTATGAGTAAGGCTAGCTACAACGAGGATCAGCCT	10-23 DNAzyme to cleave TCRβ68 transcript 7 nt upstream of the PTC
TCRβ-246	CGACTTATGAGGGCTAGCTACAACGAAATGGATCAG	10-23 DNAzyme to cleave TCRβ68 transcript 7 nt upstream of the PTC
TCRβ-250	TCAGCGACTTAGGCTAGCTACAACGAGAGTAATGGA	10-23 DNAzyme to cleave TCRβ68 transcript immediately upstream of the PTC
TCRβ-254	GCTGTCAGCGAGGCTAGCTACAACGATTATGAGTAA	10-23 DNAzyme to cleave TCRβ68 transcript 4 nt downstream of the PTC

TCRβ-257	CGTGCTGTCAGGGCTAGCTACAACGAGACTTATGAG	10-23 DNAzyme to cleave TCRβ68 transcript 7 nt downstream of the PTC
TCRβ-264	CTTTCTCCGTGGGCTAGCTACAACGATGTCAGCGAC	10-23 DNAzyme to cleave TCRβ68 transcript 14 nt downstream of the PTC
TCRβ-279	CATCAGGGATAGGCTAGCTACAACGACTCCTTTCTC	10-23 DNAzyme to cleave TCRβ68 transcript 29 nt downstream of the PTC
TCRβ-384	CCCAGTCCCGAGGCTAGCTACAACGATGCTGGCACA	10-23 DNAzyme to cleave TCRβ68 transcript 134 nt downstream of the PTC
5' RACE TCRβ Outer	AAACAAGGAGACCTTGGGTGGAGT	Primer used for TCRβ68 5' RACE experiment
5' RACE TCRβ Inner	CTCAGATCCTCGAGAACAGTCAGT	Primer used for TCRβ68 5' RACE experiment
TCRβ V+	GGGCTGAGGCTGATCCATTA	Primer used to amplify TCRβ68 cDNA fragment from cDNA for northern probe template
TCRβ C-	TGGAGTCACATTTCTCAGAT	Primer used to amplify TCRβ68 cDNA fragment from cDNA for northern probe template
GAPDH Probe+	AAGGTCGGAGTCAACGGATTTGGT	Primer used to amplify GAPDH cDNA fragment from cDNA for northern probe template
GAPDH Probe-	CATGGTTCACACCCATGACGAACA	Primer used to amplify GAPDH cDNA fragment from cDNA for northern probe template
SMG6 E+	GCGCAACTCAGAGCTGAACTG	qPCR amplification of SMG6 transcript
SMG6 E-	GGATCCTTGACAAGTTGCCTGAAC	qPCR amplification of SMG6 transcript
GAPDH C+	TGCCAAATATGATGACATCAAGAA	qPCR amplification of GAPDH transcript (internal standard)
GAPDH C-	GGAGTGGGTGTCGCTGTTG	qPCR amplification of GAPDH transcript (internal standard)
TCRβ68 SPARE RT primer	CCTTGGCACCCGAGAATTCCAAGGGAAGCCAACTCCAGAATGAGA	Primer for First strand cDNA synthesis in SPARE
SPARE Fw primer	GTTCAGAGTTCTACAGTCCGAC	Primer used for SPARE analysis
SPARE Rv primer	CCTTGGCACCCGAGAATTCCA	Primer used for SPARE analysis
SPARE P5 primer	AATGATACGGCGACCACCGACAGGTTCAGAGTTCTACAGTCCGA	Primer used for SPARE analysis
SPARE P7 index primer	CAAGCAGAAGACGGCATACGAGAT-XXXXXX-GTGACTGGAGTTCCTTGGCACCCGAGAATTCCA	Primer used for SPARE analysis; XXX XXX indicates 6 nt index sequence
RNA Oligonucleotides		
siXRN1	UUCUACGGUAAGUUCAUCUUU+AGAUGAACUUACCGUAGAAUU	siRNA for XRN1 mRNA depletion

siSMG6	CUUGUAAGUAACCUGCAGCUU+GCUGCAGGUUACUUACAAGUU	siRNA for SMG6 mRNA depletion
siUPF1	AAUGGAGCGGAACUGCAUCUUUU+AAGAUGCAGUUCCGCUCCAUUUU	siRNA for UPF1 mRNA depletion
siGL2	UCGAAGUAUUCCGCGUACGUU+CGUACGCGGAAUACUUCGAUU	control siRNA (non-specific)
SPARE 5' RNA adaptor	GUUCAGAGUUCUACAGUCCGAC	RNA adaptor for TCRβ68 SPARE analysis

SUPPLEMENTAL REFERENCES

1. Eberle, A.B., Lykke-Andersen, S., Muhlemann, O. and Jensen, T.H. (2009) SMG6 promotes endonucleolytic cleavage of nonsense mRNA in human cells. *Nat Struct Mol Biol*, **16**, 49-55.
2. Mohn, F., Buhler, M. and Muhlemann, O. (2005) Nonsense-associated alternative splicing of T-cell receptor beta genes: no evidence for frame dependence. *RNA*, **11**, 147-156.
3. Wu, L. and Belasco, J.G. (2005) Micro-RNA regulation of the mammalian lin-28 gene during neuronal differentiation of embryonal carcinoma cells. *Mol Cell Biol*, **25**, 9198-9208.
4. Boggy, G.J. and Woolf, P.J. (2010) A mechanistic model of PCR for accurate quantification of quantitative PCR data. *PLoS One*, **5**, e12355.
5. Ritz, C. and Spiess, A.N. (2008) qpcR: an R package for sigmoidal model selection in quantitative real-time polymerase chain reaction analysis. *Bioinformatics*, **24**, 1549-1551.
6. Jeong, D.H., German, M.A., Rymarquis, L.A., Thatcher, S.R. and Green, P.J. (2010) Abiotic stress-associated miRNAs: detection and functional analysis. *Methods Mol Biol*, **592**, 203-230.
7. German, M.A., Pillay, M., Jeong, D.H., Hetawal, A., Luo, S., Janardhanan, P., Kannan, V., Rymarquis, L.A., Nobuta, K., German, R. *et al.* (2008) Global identification of microRNA-target RNA pairs by parallel analysis of RNA ends. *Nat Biotechnol*, **26**, 941-946.

8. German, M.A., Luo, S., Schroth, G., Meyers, B.C. and Green, P.J. (2009) Construction of Parallel Analysis of RNA Ends (PARE) libraries for the study of cleaved miRNA targets and the RNA degradome. *Nat Protoc*, **4**, 356-362.

9. Jeong, D.H. and Green, P.J. (2012) Methods for validation of miRNA sequence variants and the cleavage of their targets. *Methods*, **58**, 135-143.

10. Langmead, B., Trapnell, C., Pop, M. and Salzberg, S.L. (2009) Ultrafast and memory-efficient alignment of short DNA sequences to the human genome. *Genome Biol*, **10**, R25.

11. Martin, M. (2011) Cutadapt removes adapter sequences from high-throughput sequencing reads. *EMBnet.journal*, **17**, 10-12.

12. Kim, D., Pertea, G., Trapnell, C., Pimentel, H., Kelley, R. and Salzberg, S.L. (2013) TopHat2: accurate alignment of transcriptomes in the presence of insertions, deletions and gene fusions. *Genome Biol*, **14**, R36.

13. Trapnell, C., Roberts, A., Goff, L., Pertea, G., Kim, D., Kelley, D.R., Pimentel, H., Salzberg, S.L., Rinn, J.L. and Pachter, L. (2012) Differential gene and transcript expression analysis of RNA-seq experiments with TopHat and Cufflinks. *Nat Protoc*, **7**, 562-578.

14. Huang da, W., Sherman, B.T. and Lempicki, R.A. (2009) Systematic and integrative analysis of large gene lists using DAVID bioinformatics resources. *Nat Protoc*, **4**, 44-57.

15. Crooks, G.E., Hon, G., Chandonia, J.M. and Brenner, S.E. (2004) WebLogo: a sequence logo generator. *Genome Res*, **14**, 1188-1190.

16. Rice, P., Longden, I. and Bleasby, A. (2000) EMBOSS: the European Molecular Biology Open Software Suite. *Trends Genet*, **16**, 276-277.
